# Approximation to pain‐signaling network in humans by means of migraine

**DOI:** 10.1002/hbm.25261

**Published:** 2020-10-28

**Authors:** Jonas Aurel Hosp, Marco Reisert, Charlotte von Kageneck, Michel Rijntjes, Cornelius Weiller

**Affiliations:** ^1^ Faculty of Medicine, Department of Neurology and Neuroscience Medical Center – University of Freiburg Freiburg Germany; ^2^ Faculty of Medicine, Department of Stereotactic and Functional Neurosurgery University of Freiburg Freiburg Germany; ^3^ Department of Medical Physics Freiburg University Medical Center Freiburg Germany

**Keywords:** global tractography, humans, migraine, pain matrix

## Abstract

Nociceptive signals are processed within a pain‐related network of the brain. Migraine is a rather specific model to gain insight into this system. Brain networks may be described by white matter tracts interconnecting functionally defined gray matter regions. Here, we present an overview of the migraine‐related pain network revealed by this strategy. Based on diffusion tensor imaging data from subjects in the Human Connectome Project (HCP) database, we used a global tractography approach to reconstruct white matter tracts connecting brain regions that are known to be involved in migraine‐related pain signaling. This network includes an ascending nociceptive pathway, a descending modulatory pathway, a cortical processing system, and a connection between pain‐processing and modulatory areas. The insular cortex emerged as the central interface of this network. Direct connections to visual and auditory cortical association fields suggest a potential neural basis of phono‐ or photophobia and aura phenomena. The intra‐axonal volume (V_intra_) as a measure of fiber integrity based on diffusion microstructure was extracted using an innovative supervised machine learning approach in form of a Bayesian estimator. Self‐reported pain levels of HCP subjects were positively correlated with tract integrity in subcortical tracts. No correlation with pain was found for the cortical processing systems.

## Graphical abstract

Brain networks may be described by white matter tracts interconnecting functionally defined grey matter regions. Here, we present an overview of the migraine‐related pain network revealed by this strategy. Based on diffusion tensor imaging data from subjects in the Human Connectome Project database, we used a global tractography approach to reconstruct white matter tracts connecting brain regions that are known to be involved in migraine‐related pain signaling.

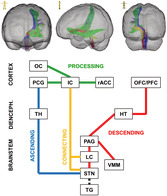

## 1 INTRODUCTION

Pain signals are processed within a network which consists of the following elements (Goadsby et al., 2017; Legrain, Iannetti, Plaghki, & Mouraux, 2011; Schnitzler & Ploner, 2000): (a) a sensory discriminative pathway that sends nociceptive signals to the sensory cortex, (b) a cortical network that assesses and evaluates nociceptive stimuli, and (c) a descending pathway (DP) that modulates the perception and transduction of pain signals. These available models are based on a patchwork of animal studies as well as activation patterns derived from functional imaging in humans (Noseda & Burstein, 2013; Pietrobon & Moskowitz, 2013) that were focused on gray matter regions (e.g., brainstem nuclei, subcortical nuclei, or cortical regions) involved in pain signaling.

However, different types of pain and pain disorders rely on different (patho)physiological mechanisms and may involve different pain‐processing centers (for review, see (Yam et al., 2018)). Thus, pain‐specific approaches need to be established for particular pain‐related disorders. Migraine is a primary headache disorder that has a prevalence of around 12–16% (Smitherman, Burch, Sheikh, & Loder, 2013) and has been ranked as the third‐highest cause of disability worldwide in males and females under 50 years of age (Steiner, Stovner, & Vos, 2016). Migraine headache is a recurrent headache disorder manifesting in attacks lasting 4–72 hr. Typical characteristics of the headache are unilateral location, pulsating quality, moderate, or severe intensity, aggravation by routine physical activity and association with nausea and/or photophobia and phonophobia (Headache Classification Committee of the International Headache Society (IHS), 2018). Migraine is furthermore characterized by different states (acute attack vs. interictal state; (Schulte et al., 2020)), phenotypes (e.g., migraine with and without aura; (Goadsby et al., 2017)) and long‐term effects due to chronification (e.g., sporadic vs. chronic migraine; (Planchuelo‐Gómez et al., 2020a; Planchuelo‐Gómez et al., 2020b)).

Due to its high prevalence and socioeconomic impact (Steiner et al., 2016), migraine ranks among the most well‐investigated pain disorders (Charles, 2018). Since the original study identifying brain stem structures being involved in human migraine attacks (Weiller et al., 1995), numerous studies have clearly described cortical and subcortical gray matter related to migraine (e.g., Schulte et al., 2020; Schulte & May, 2016). As migraine affects white matter structure as well (Chong et al., 2019; Chong & Schwedt, 2015) it can be assumed that these regions are interconnected and functionally interact with each other. Thus, migraine seems a specified model to gain further insight into the network and the basic mechanisms how the brain processes pain signals.

However, a network is defined by its bars and its nodes. Here we present a novel, white matter architecture‐based approach to identify the connecting bars of the migraine‐related pain signaling network. We use gray matter regions of interest (ROIs) involved in migraine‐related pain signaling as network nodes to perform global tractography based on diffusion‐tensor imaging (DTI). For selection of ROIs, we did not focus on anatomical regions that are differentially involved in particular states or phenotypes of migraine. We focused on regions that are robustly involved under various conditions of this complex pain disorder to depict the “common denominator” of the migraine‐related central pain‐processing system.

The resulting network covers the ascending (trigeminothalamic) pathway, the cortical processing network, the modulating DP, and a connection between the cortical system and the DP. The insular cortex (IC) turned out to be a central interface and junctional point in this system. To demonstrate that the reconstructed tracked fiber bundles are related to the processing of nociceptive signals, the intra‐axonal volume (V_intra_)—a measure of functional fiber integrity—was extracted from binary tract masks using a supervised machine learning approach in the form of a Bayesian estimator. V_intra_ was then correlated to a general numerical pain‐rating scale. The ascending, descending and connecting—but not the cortical processing systems—were each correlated to pain experience. Importantly, as we used DTI sequences from a cohort of 100 subjects derived from the Human Connectome Project (HCP) database, we did not study migraine‐associated changes in migraineurs. However, our work may lay a basis for dedicated research on the role of white matter connections in various different aspects of migraine or migraine treatment.

## 2 MATERIALS AND METHODS

### 2.1 Subjects and magnetic resonance imaging

We used diffusion data derived from 100 subjects entered into the HCP (Q1, S3) data corpus (resolution 1.25 mm isotropic, three b‐shells with 1,000; 2,000; 3,000—for more details on the protocol and preprocessing, see Glasser et al., 2013), mean age 29 ± 3.7; 64 females, 36 males). For mapping into MNI template space, all of the accompanying T1 weighted images were subjected to CAT12 segmentation/normalization. For normalization to MNI space, we used the deformation fields provided by CAT12. All study procedures involving human participants were performed in accordance with the ethical standards of the institutional and/or national research committee, and with the 1964 Helsinki declaration and its later amendments, or comparable ethical standards. Please refer to the HCP homepage for more information (https://www.humanconnectome.org).

### 2.2 General research strategy

To define fiber tracts belonging to the migraine‐related pain network, we conducted the following approach:

1. Key anatomical structures involved in migraine‐associated pain perception, processing and modulation were identified via a literature search, and the relevant ROIs were defined in MNI space.
2. Global tractography was performed individually for each subject in an unconstrained manner. The above‐defined ROIs were then warped from MNI to native space using the deformation fields provided by CAT12. The streamlines of the global connectome were selected according to the strategy described below.
3. Fiber density maps and directional fiber density maps were computed for each of the bundles selected. After rendition in native space, density maps were normalized to template space and aggregated to yield a group density representation of the bundles in MNI standard space.
4. The intra‐axonal volume (V_intra_) that was derived from a three‐compartment diffusion model (Reisert, Kellner, Dhital, Hennig, & Kiselev, 2017) was computed at each identified bundle location and correlated with self‐reported experience of pain, as measured by the NIH Toolbox Pain Intensity Survey (NTPIS; Cook et al., 2013). The details for each of these processing steps are described below.

### 2.3 Definition of ROIs

**FIGURE 1:**
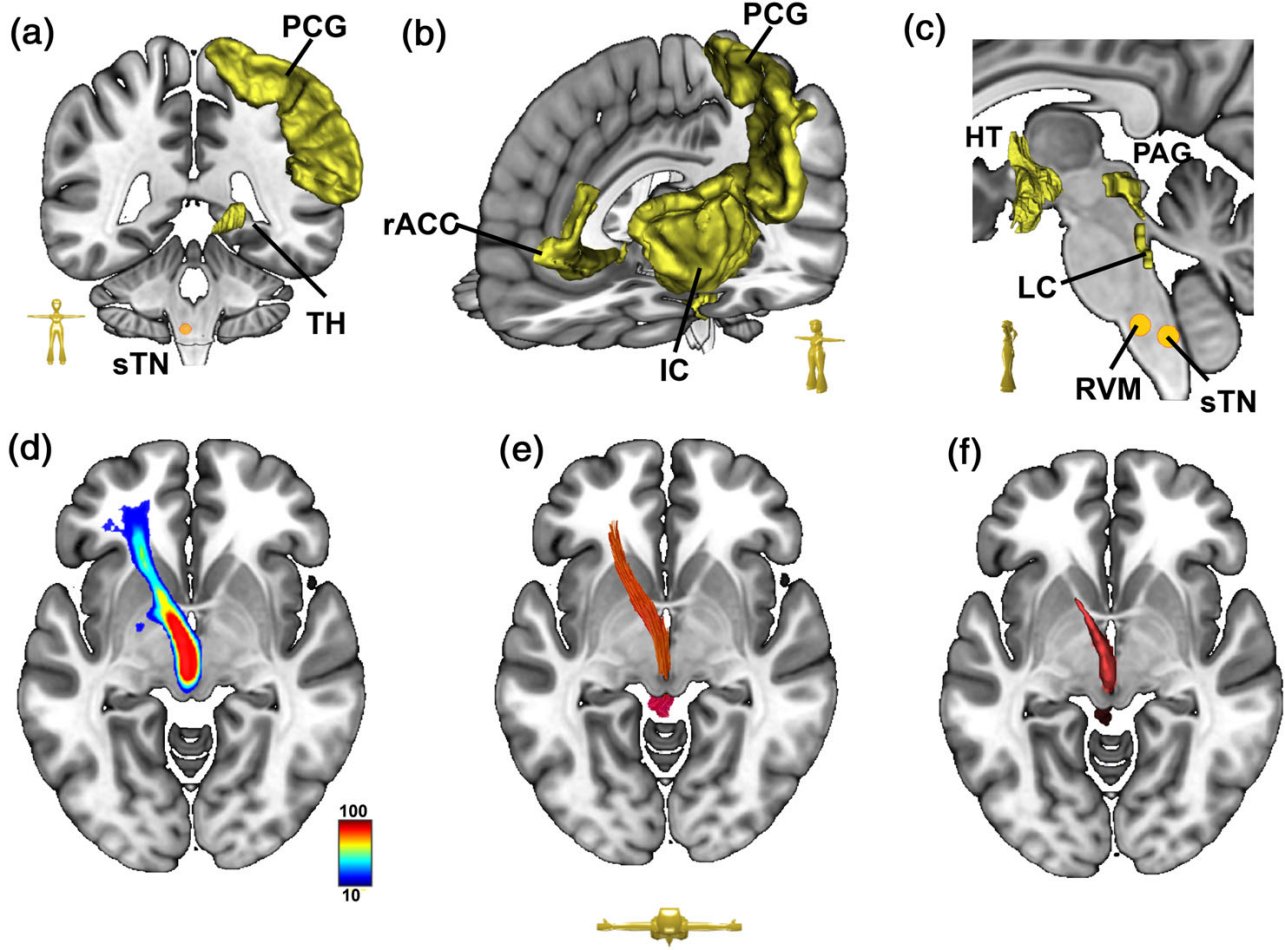
Reconstruction of migraine‐associated tracts. (a) Regions of interest (ROIs) of the ascending pathway (AP) related to pain perception: spinal trigeminal nucleus (sTN); thalamus (TH; ventral posterolateral nucleus); postcentral gyrus (PCG). (b). ROIs of the pain‐processing network: PCG; insular cortex (IC); rostral anterior cingulate cortex (rACC). (c). ROIs of the ascending system related to pain modulation: sTN; rostral ventromedial medulla (RVM); locus coeruleus (LC); periaqueductal gray (PAG); and hypothalamus (HT). (d) An example of group density representation in the MNI standard space. The color code represents the voxel‐wise probability (in %) of streamline occurrence based on individual fiber bundles. (e) Deterministic bundle‐specific tractography based on the tensor field of the tract shown in (d). (f) An enveloping mask covering the core of the tract shown in (d) was generated based on the probability threshold (i.e., >80%)

The key structures involved in migraine‐associated pain perception, processing and modulation were defined by a literature search using the NIHS Public Archive for the Refereed Literature (PUBMED; https://www.nvbi.nlm.nih.gov). The following search criteria were used: migraine AND MNI; migraine AND MRI; pain matrix; ascending pain system; descending pain system; animal model AND migraine. Relevant publications were selected and analyzed (Supplementary Table S1). Migraine however is a complex disorder that is characterized by different states (acute attack vs. interictal state; (Schulte et al., 2020)), phenotypes (e.g., migraine with and without aura; (Goadsby et al., 2017)) and long‐term effects due to chronification (sporadic vs. chronic migraine; (Planchuelo‐Gómez et al., 2020b)). Each particular condition may rely on different mechanisms and structures. As it was our aim to visualize this rough scaffold of white matter connections harboring the flow of pain‐related signals within the brain, we focused on regions that are robustly involved in various conditions and aspects of migraine headache to depict the “common denominator” of the migraine‐related central pain‐processing system. These ROIs were then compared and checked against ROIs derived from the literature on general pain processing (Supplementary Table S2). We finally selected the following ROIs that, according to the actual literature, are robustly involved into the processing of migraine headache and general processing of pain (Figure 1a–c): the spinal trigeminal nucleus (sTN; seed ±4 −42, −55; radius 4 mm; (Schulte & May, 2016)); the rostral ventromedial medulla (RVM; seed 0 −36, −51; radius 4 mm; adapted from (Mills et al., 2018)); the periaqueductal gray (PAG; mask adapted from (Edlow et al., 2012)); the locus coeruleus (LC; mask adapted from (Edlow et al., 2012)); hypothalamus (HT; mask adapted from (Ilinsky et al., 2018)); the postcentral gyrus (PCG; mask adopted from the Desikan–Killiany atlas, (Kanaan, Allin, Picchioni, Shergill, & McGuire, 2016)), which harbors the somatosensory cortex (Brodmann areas S1–3; (Brodmann, 1909)); the ventral posterolateral and ‐medial thalamic nucleus (TH; mask adapted from (Ilinsky et al., 2018)); the IC (mask adapted from the Desikan–Killiany atlas, (Kanaan et al., 2016)); the rostral anterior cingulate cortex (rACC; mask adapted from the Desikan–Killiany atlas, (Kanaan et al., 2016)). As a literature‐based selection of ROIs is arbitrary to a certain degree, literature research and subsequent selection of ROIs was performed by researcher (C. K.) that was not involved in the further process of global fiber tracking.

### 2.4 Global tractography algorithm

White‐matter probability maps obtained from CAT12 were thresholded at a probability of 0.5 to determine the area of fiber reconstruction. For tractography analysis, we followed the global approach described by Reisert et al. (2011). As opposed to local walker‐based tractography, global fiber tracking aims to find a fiber configuration that delivers the best explanation for the acquired diffusion‐weighted MRI (dMRI) data. The optimization process is essentially similar to a polymerization process: the streamlines are initially short and fuzzy, whereas during the optimization phase, the connections proliferate, and fibers become increasingly congruent with the data. The algorithm is based on so‐called “simulated annealing.” The system is initially set at a relatively high temperature, which is slowly decreased during iterations to progressively obtain more accurate results. Global fiber tractography is usually found to be less sensitive to noise, and fiber density is directly related to the measured data itself. From the two parameter sets provided by the toolbox (Reisert et al., 2011), we chose the “dense” preset for our analyses. In addition, to increase reproducibility, we increased the number of fibers using the following accumulation strategy: after the cooling‐down phase, the temperature was again increased to 0.1, and the state further iterated for 10^7^ iterations. This procedure was iterated over five rounds and the tracts resulting from each round were accumulated to obtain one final tractogram that was five times larger than the initial one. This approach was proposed by Schumacher et al. (2018) and showed a much higher retest reliability.

An exhaustive approach was conducted to define migraine‐associated tracts: ROIs were applied in all possible combinations for fiber selection. Combinations of ROIs that were not connected—that is, those that did not yield any interlinking streamline—were excluded from all further investigations. By definition, a streamline connects a combination of ROIs if any of its supporting points lies within each ROI.

### 2.5 Fiber density generation and aggregation

Fiber densities were computed by means of trilinear interpolation on an isotropic matrix with 1.5 mm resolution. Before averaging, the densities were thresholded at a cut‐off value of 1 mm streamline length per voxel. Group averages of the streamline indicator images were built, and a color‐coded scheme was used to indicate the voxel‐wise probability (in %) of fiber streamline occurrence in the entire group. To understand the true extension of the tracts, the structure was overlaid onto a T1W template in MNI space (Figure 1d). Directional fiber density maps were then obtained by rendering the rank‐1 tensor formed by the tangent of the fibers. The tensor field representation allowed the calculation of mean values in the common additive manner, as for the scalar densities. The directional density maps were normalized as explained above. However, since the tensorial nature of the field had to be taken into account for normalization to MNI standard space, we used the Jacobian matrix of the associated template warp to map the tensor from subject space to MNI standard space. The resulting tensor fields in MNI standard space were then used for deterministic bundle‐specific tractography (Figure 1e). This was obtained by randomly placing seeds in high‐density regions (threshold >10^−1^), with a very loose stopping criterion (threshold >10^−8^) to allow inclusion of cortical areas. This streamlining algorithm is similar to the commonly used FACT algorithm (Mori, Crain, Chacko, & van Zijl, 1999; Mori & van Zijl, 2002). The medical imaging platform NORA was used for visualization and bundle specific tractography (www.nora-imaging.org). The resulting tracts varied somewhat between subjects in terms of robustness and reproducibility, as indicated by the low voxel‐wise probability of streamline occurrence. A low probability of fiber occurrence can hence be explained by either a high degree of interindividual variability in tract anatomy, a low absolute number of streamlines, or a combination of both factors. To exclude tracts that were especially weak or variable, only those that included voxels with ≤40% probability of streamline occurrence were taken into account.

### 2.6 Generation of tract masks and extraction of dMRI‐parameters

To evaluate microstructural diffusion parameters, masks covering the core of a particular tract were created using a binary threshold approach (Figure 1f). Threshold values were defined based on probability maps: only voxels with a certain probability of streamline occurrence (i.e., “lower limit,” Supplementary Table S2) were included in the analysis. The “upper limit” was defined by the voxel showing the maximal probability of streamline occurrence. Masks were then warped into subject space for extraction of the dMRI parameter V_intra_. The dMRI parameters are based on a standard white matter model (Novikov, Jensen, Helpern, & Fieremans, 2014; Novikov, Reisert, & Kiselev, 2018; Reisert et al., 2017). This is a three‐compartment model that distinguishes between V_intra_, extra‐axonal volume (V_extra_), and the free cerebrospinal fluid environment. These parameters have been extracted using a supervised machine learning approach in the form of a Bayesian estimator (for details, see (Reisert et al., 2017)). Our approach shares similarities with the neurite orientation dispersion and density imaging (NODDI) technique (Zhang, Schneider, Wheeler‐Kingshott, & Alexander, 2012), which relies on maximum likelihood estimation. However, while NODDI requires stabilization by a priori constraints, our approach is able to keep the unconstrained microstructural tissue models while still allowing the determinability of model parameters to be estimated explicitly. Among the mesoscopic dMRI parameters extracted by our Bayesian approach, the best measure of functional fiber integrity turned out to be the V_intra_ that corresponds to the axonal water fraction (AWF) of NODDI. Indeed, a reduction in V_intra_ was the most sensitive measure of white matter damage in the cervical spinal cord (By, Xu, Box, Bagnato, & Smith, 2017) and supratentorial fiber tracts (Margoni et al., 2019) of patients suffering from multiple sclerosis. In contrast, increased intra‐neurite volume within the gray matter was associated with higher intelligence (Genç et al., 2018).

Importantly, no information about the occurrence of migraine, headache, or other pain disorders was available for our cohort. However, recent pain experiences were self‐reported by HCP subjects and assessed by the NTPIS (Cook et al., 2013). In this survey, subjects rate their average pain level over a 7‐day period on a numerical scale of 0 to 10, where 0 = no pain, and 10 = worst pain. There was, however, no further information on pain modality.

To assess the correlation between functional fiber integrity (measured by V_intra_) and the average pain level (measured by NTPIS score), we calculated a nonparametric multiple linear regression model with the factors “age” and “gender” as covariates. To correct for multiple comparisons, a strict (Bonferroni correction) and a less conservative (correction for a false discovery rate (FDR) <5%, threshold log(p) = −1.565995) variant were calculated.

## 3 RESULTS

To define fiber tracts that contribute to the migraine‐related pain network, a literature search was first performed, whereby key anatomical structures involved in migraine‐associated pain perception, processing, and modulation were identified (Supplementary Tables S1 and S2). The corresponding ROIs in the MNI space were then selected (Figure 1a–c). Based on a normative connectome in MNI space (*n* = 100 subjects; HCP database, www.humanconnectome.org), combinations of interconnected ROIs were defined using an unconstrained, random approach. Resulting combinations of interconnected ROIs were then warped from MNI to native space to select streamlines based on each subject's individual connectome. The resulting fiber bundles were then re‐warped in MNI standard space to compute group density representations (Figure 1d). Based on the resulting tensor fields, deterministic bundle‐specific tractography was performed to visualize fiber tracts (Figure 1e). Masks enveloping the core of each tract were then generated according to a defined threshold (probability of streamline occurrence, Figure 1f). Masks were warped from MNI to native space to extract the mesoscopic diffusion measure V_intra_, which was subsequently correlated with the self‐reported experience of pain.

Based on literature, several subsystems of the pain‐processing system can be differentiated (Goadsby et al., 2017; Legrain et al., 2011; Schnitzler & Ploner, 2000): (a) a sensory discriminative pathway that sends nociceptive signals to the sensory cortex, (b) a cortical network that assesses and evaluates nociceptive stimuli, and (c) a DP that modulates the perception and transduction of pain signals. The tracts resulting from our tracking approach fitted perfectly into this arrangement. Thus, we decided to adopt this classification for the presentation of our results. However, a fourth pathway mediating a connection between the insula and brainstem centers had to be added.

**FIGURE 2:**
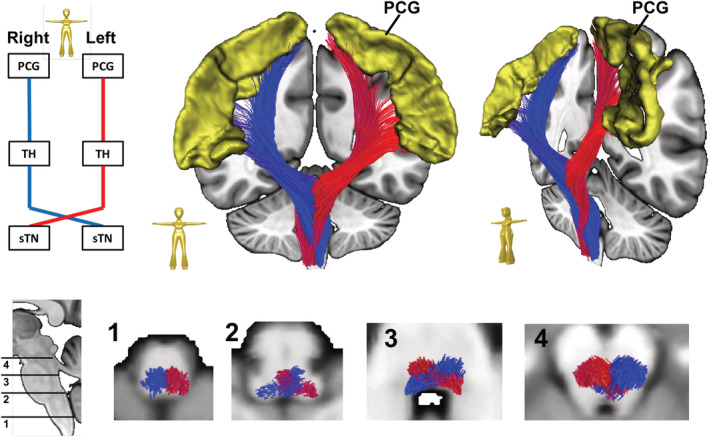
The ascending/nociceptive pathway (AP): Top, left panel. Scheme showing the regions of interest (ROIs) used for streamline selection. Top, right panel. An overview of the AP produced by deterministic bundle‐specific tractography: streamlines are attached to the spinal trigeminal nucleus (sTN) and cross the midline at the brainstem level. Streamlines project from the ventral posterolateral nucleus of the thalamus (TH) to reach the postcentral gyrus (PCG). Note the prominent innervation of the suprasylvic portion of the PCG containing sensory representation of head and face. Bottom. Higher magnification of axial brainstem sections at various levels reveals the crossing points of different somatosensory systems: whereas protopathic fibers (nociception/thermesthesia) cross the midline close to the sTN at the level of the lower pons (2), epicritic fibers (proprioception) cross the midline at the level of the mesencephal lemniscal decussation (4)

**FIGURE 3:**
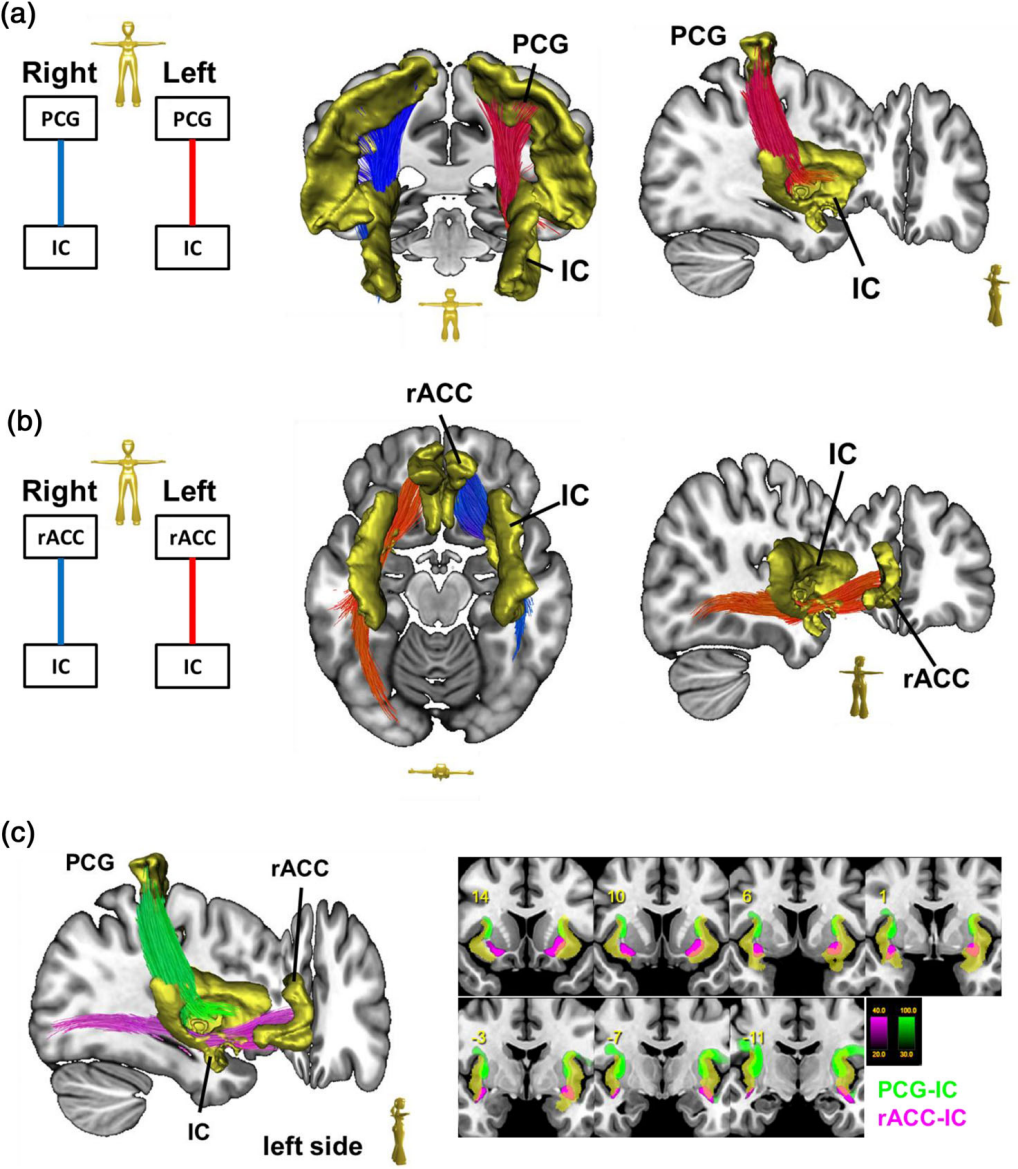
The processing network (PN): (a) left panel. Scheme showing the regions of interest (ROIs) used for streamline selection. An overview of the connection between the postcentral gyrus (PCG) and the insular cortex (IC) produced by deterministic bundle‐specific tractography. (b) ROIs that were used for streamline selection are indicated by the scheme. An overview of the connection between the rostral anterior cingulate cortex (rACC) and the IC, based on deterministic bundle‐specific tractography. The left rACC‐IC tract is more pronounced than that on the right, and streamlines passing through the IC project toward the auditory and visual association cortices. (c) Projections from the PCG and rACC are characterized by specific termination fields: fiber connections between the insula and rACC (magenta) and the insula and PCG (green) (shown in (a) and (b)) are indicated by bundle‐specific tractography (left) and axial density representation maps in MNI space (right). The color code represents the probability (in %) of fiber streamline occurrence in the entire group. Yellow shading denotes the insular cortex (IC). The PCG is connected to the posterodorsal insula, while the rACC is connected to the anteroventral portion of the IC

**FIGURE 4:**
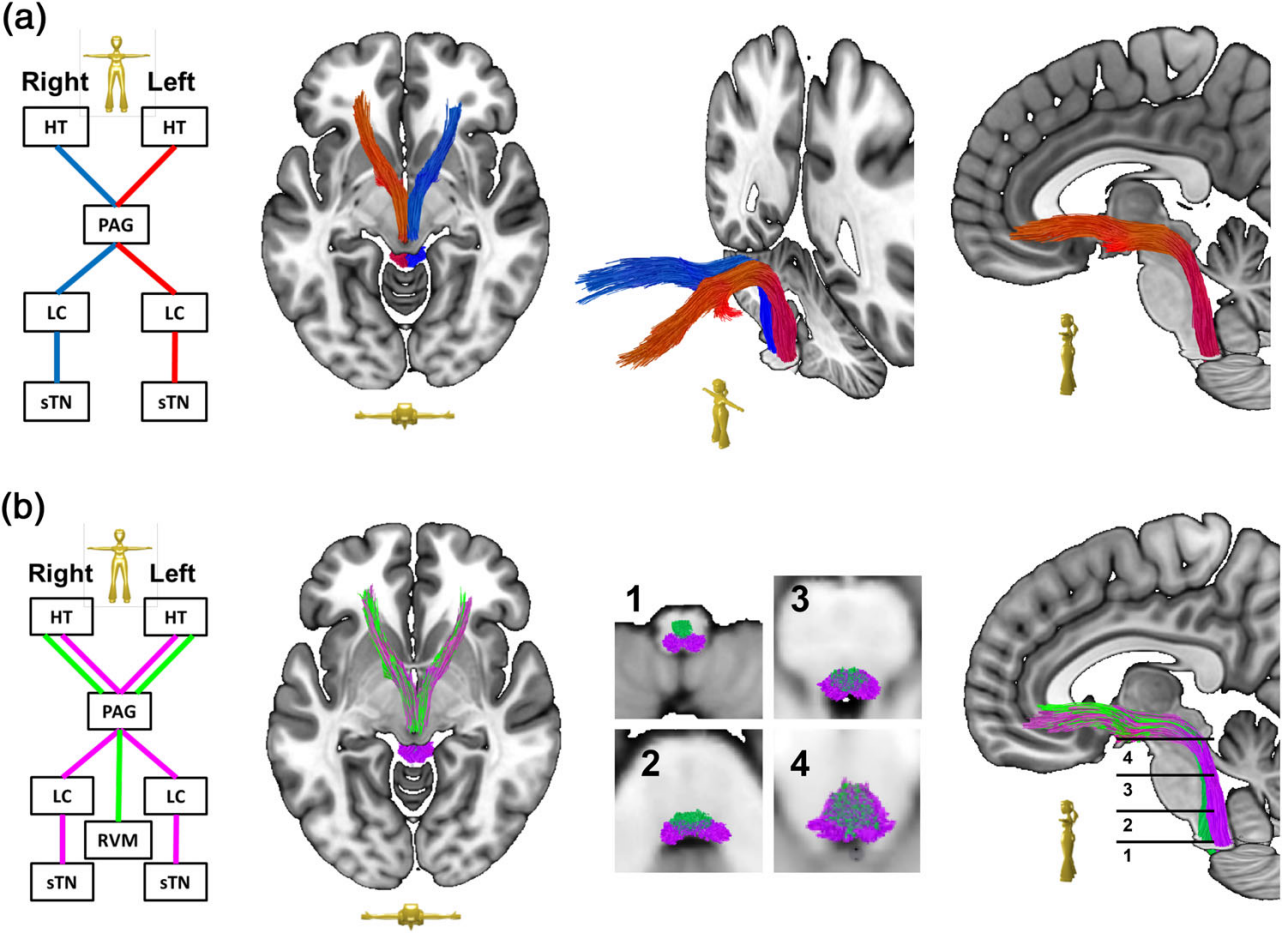
The descending pathway (DP): (a) Scheme showing the regions of interest (ROIs) used for streamline selection. An overview of the DP main trunk produced by deterministic bundle‐specific tractography. The pathway connects the hypothalamus (HT), the periaqueductal gray (PAG), the locus coeruleus (LC), and the spinal trigeminal nucleus (sTN). Streamlines can be further tracked to the prefrontal and orbitofrontal cortices. A residual portion of streamlines was falsely attracted by the anterior commissure. (b) An unpaired ventral column (green) could be superimposed onto the dorsal part of the DP (magenta) (also shown in (a)). This ventral column connects the rostral ventromedial medulla (VMM) to the PAG without passing through the LC. Above the level of the PAG, both DP columns are inseparably intermingled

**FIGURE 5:**
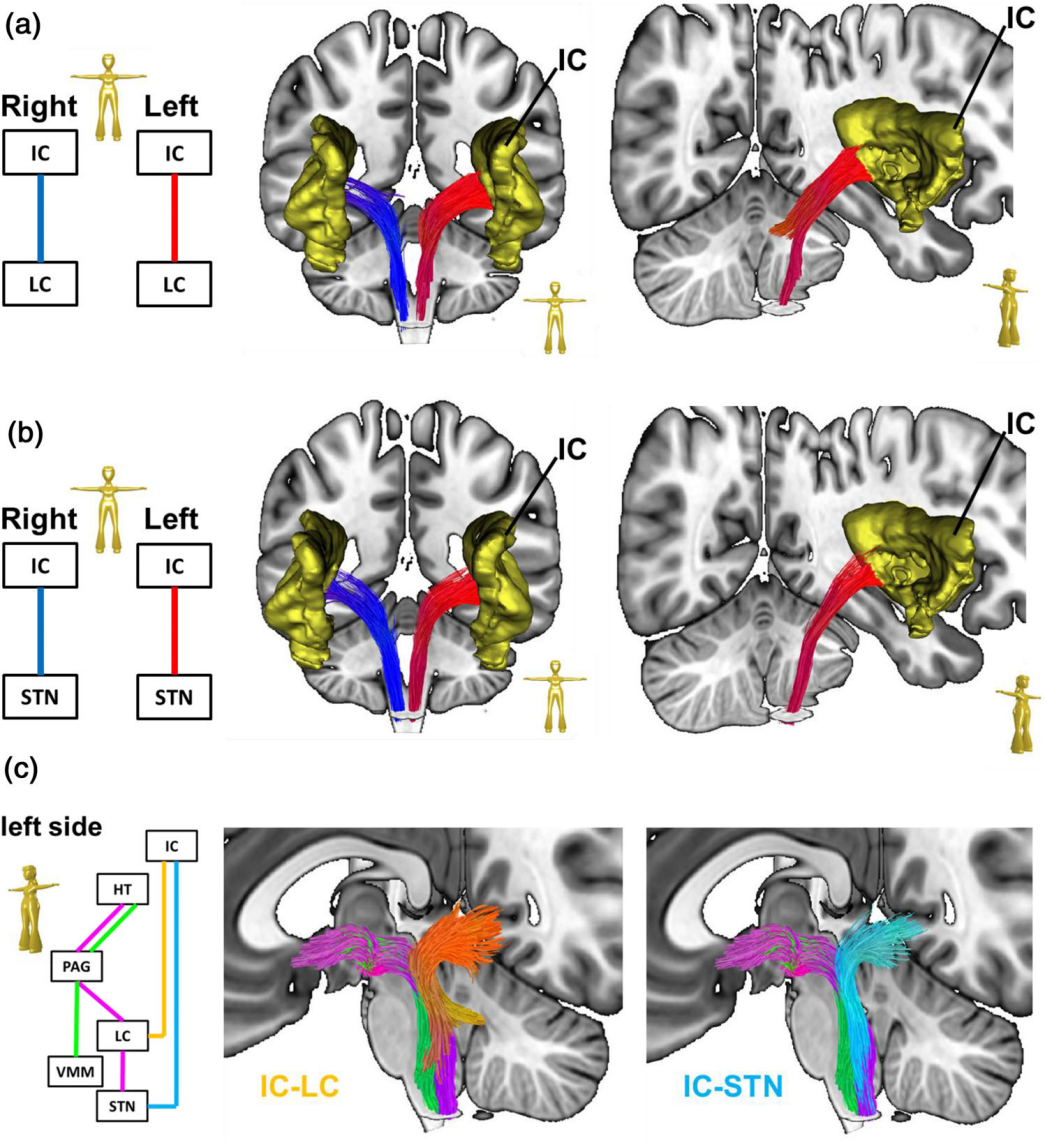
The connecting system (CS) between the processing network and descending pathways: (a) left panel. Scheme showing the regions of interest (ROIs) used for streamline selection. The pathway connects the insular cortex (IC) to the locus coeruleus (LC). Right panel. An overview of the entire IC‐LC tract produced by deterministic bundle‐specific tractography. Streamlines could be further tracked to the ipsilateral cerebellum. (b) In addition to the IC‐LC tract, a pathway connecting the IC to the spinal trigeminal nucleus (sTN) was identified. (c) The topographical relationship between the dorsal/ventral columns of the DP and the IC‐LC and IC‐sTN tracts, respectively, is displayed for the left side, based on deterministic bundle‐specific tractography. Both tracts originate from the dorsoventral portion of the IC. The IC tract appears to extend into the dorsal and ventral column, whereas the IC‐sTN tract is confined to the dorsal column of the DP

**FIGURE 6:**
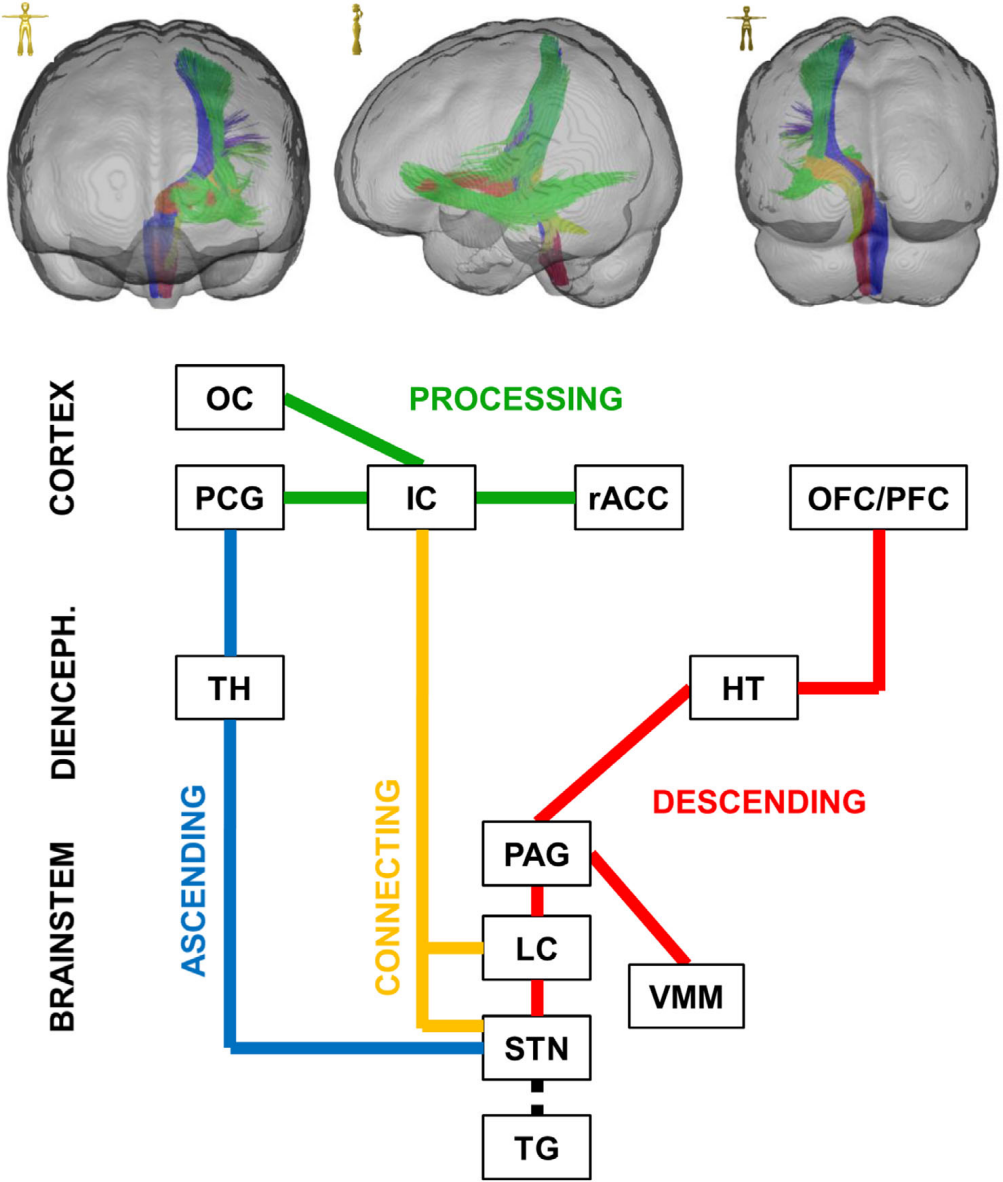
The migraine‐related pain‐signaling network: Top. Three‐dimensional representation of the left‐sided migraine‐related pain‐signaling network based on the deterministic bundle‐specific tractographies shown in Figures 2, 3, 4, 5. Color‐coding indicates the different systems: ascending pathway (AP)—blue; processing network (PN)—green; descending pathway (DP)—red; connecting system (CS)—ocher. Bottom: Organigram of the migraine‐related pain‐signaling network using a similar color‐coding scheme. The insular cortex (IC) turned out to be the central hub of the system. CB, cerebellum; IC, insular cortex; HT, hypothalamus; LC, locus coeruleus; OC, occipital cortex; OFC, orbitofrontal cortex; PAG, periaqueductal gray; PCG, postcentral gyrus; PFC, prefrontal cortex; rACC, rostral anterior‐cingulate cortex; sTN, spinal trigeminal nucleus; TC, temporal cortex; TH, thalamus; TG, trigeminal ganglion; VMM, rostral ventromedial medulla

**FIGURE 7:**
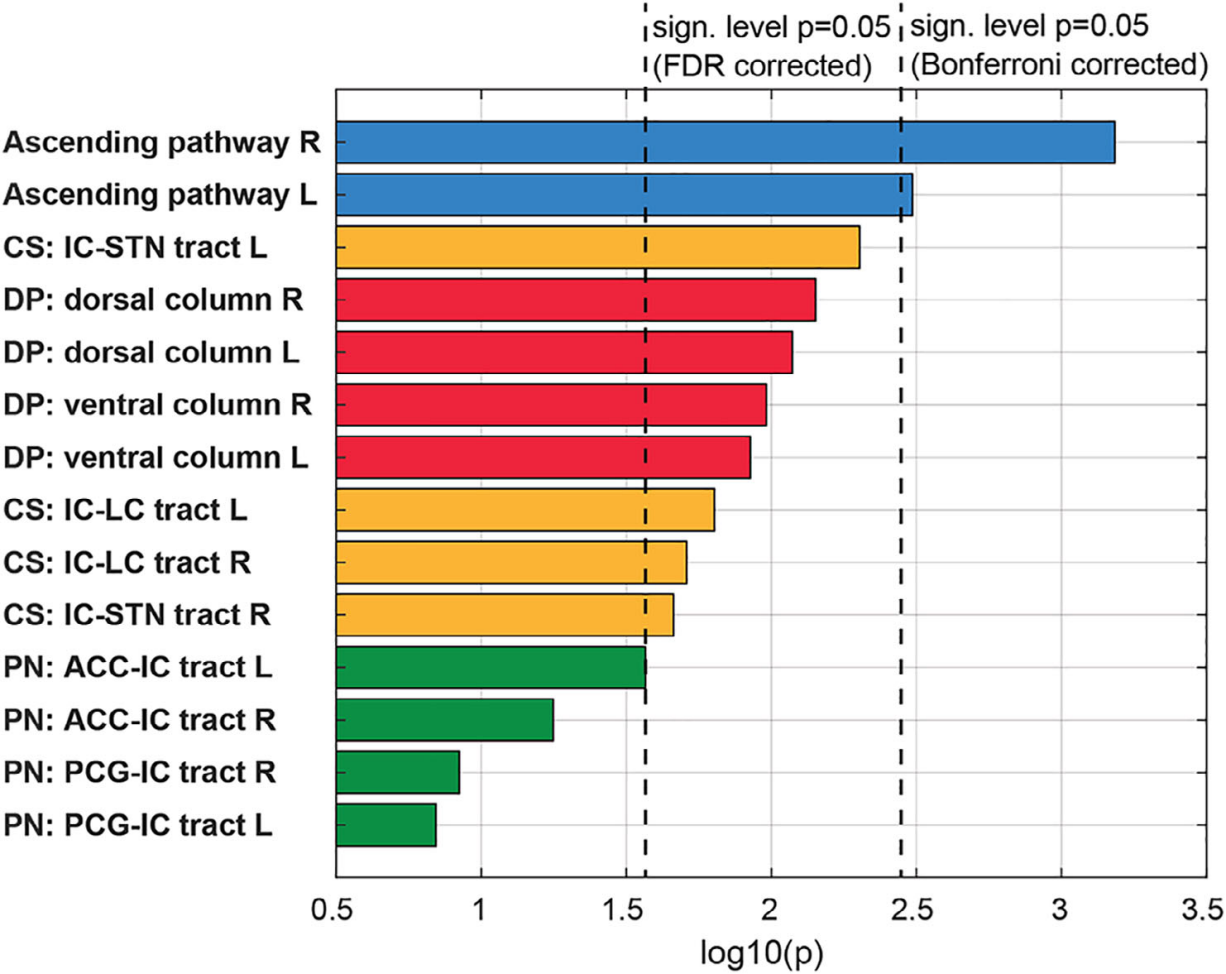
Correlation between functional fiber integrity and self‐reported pain levels: Self‐reported pain data as recorded by the NIH Toolbox Pain Intensity Survey (NTPIS) was correlated with the intra‐axonal volume fraction (V_intra_) using a nonparametric multiple linear regression model. Color‐coding indicates the different systems: ascending pathway (AP)—blue; processing network (PN)—green; descending pathway (DP)—red; connecting system (CS)—ocher. After Bonferroni's correction for multiple comparisons, the left and right APs showed a significant correlation with each other. Using the less conservative correction for a false discovery rate (FDR) <5%, the left and right AP, DP, and CS tracts showed a significant correlation. There was no significant correlation between NTPIS and V_intra_ for the tracts of the PN

1. The ascending/nociceptive pathway (AP): The ascending pathway (AP) delivers nociceptive information from the head/face region toward the primary somatosensory fields in the PCG (Kaas et al., 1979; Kenshalo & Isensee, 1983). Streamlines were hence attached to the sTN, the contralateral sensory thalamus (TH, posterior ventral posterolateral nucleus), and the PCG (Figure 1a). An overview of the entire pathway obtained from deterministic bundle‐specific tractography is displayed in Figure 2, top. Interestingly, in addition to the nociceptive protopathic pathway that crosses the midline soon after leaving the sTN, epicritic (proprioceptive) fibers that do not cross the midline until the point of the mesencephalic‐lemniscal decussation could be observed (Figure 2, bottom). The group density representation maps underlying the bundle‐specific tractography of the AP are presented in Supplementary Figure S1.
2. The cortical processing network (PN): Among the cortical structures involved in the processing of migraine‐associated signals, we identified three regions of particular interest (Figure 1b): (a) the PCG, which contains the cortical representation field for nociceptive input, (b) the IC, and (c) the rACC. Here, two of the tracts connecting the PCG and rACC to the IC were identified as the PCG‐IC tract and the rACC‐IC tract. Figure 3a shows the PCG‐IC using tract deterministic bundle‐specific tractography. The underlying group density representation maps are shown in Supplementary Figure S2. Figure 3b shows the rACC‐IC tract in similar deterministic bundle‐specific tractography. The left‐sided tract is more pronounced than the right‐sided. Some fibers pass through the IC and project toward the auditory (superior and medial temporal gyrus) and visual (middle occipital gyrus) association cortices. The underlying group density representation maps are displayed in Supplementary Figure S3. Figure 3c ultimately displays the topographical distribution of PCG and rACC afferents within the IC; whereas the rACC‐IC tract is connected to the anteroventral portion of the IC, the PCG‐IC tract shows a broad insertion zone, with focus on the posterodorsal insula.
3. The DP: The DP is a complex pain modulatory network that includes several subcortical regions (Figure 1c): the HT, the PAG, the LC, the RVM, and the sTN. Interestingly, all ROIs except for the RVM were marked with a robust tract running from the orbitofrontal and frontopolar cortices, over the HT, and along the dorsal brainstem, thereby connecting the PAG, LC, and sTN. Figure 4a presents an overview of this “dorsal column.” In addition, an unpaired “ventral column” that directly connects the RVM with the PAG, and then unites with the dorsal column of DP, was defined. Figure 4b shows an overview of the “ventral column” of the DP superimposed onto the “dorsal column” The underlying group density representation maps are shown in Supplementary Figures S4 and S5.
4. The connecting system (CS): The AP and PN (PN) are connected via the PCG‐IN tract (Figure 3a). The DP, which serves as the “efferent limb” of the migraine‐associated pain network, is coupled to the PN via two fiber tracts that form the CS and include the following ROIs (Figure 1b,c): the IC, the LC, and the sTN. Figure 5a shows the IC‐LC tract that was identified using tract‐deterministic bundle‐specific tractography. The underlying group density representation maps are shown in Supplementary Figure S6. Interestingly, the IC‐LC tract is characterized by a prominent branch targeting the ipsilateral cerebellum. Figure 5b shows the IC‐sTN tract in similar deterministic bundle‐specific tractography. Underlying group density representation maps are shown in Supplementary Figure S7. Figure 5c ultimately displays the topographical relationship between both CS‐tracts and the dorsal and ventral columns of the DP: both tracts originate from the dorsoventral portion of the IC. The IC‐tract appears to extend into the dorsal and ventral column, whereas the IC‐sTN tract is confined to the dorsal column of the DP.
5. The migraine‐related pain signaling network—organigram and correlation to pain experience: Figure 6 provides an overview of the migraine‐related pain‐signaling network. The IC turned out to be a central hub, as it served as an interface for all of the subsystems defined in Figures 2, 3, 4, 5. Furthermore, as a part of the PN, the IC is connected to the anterior cingulum and the occipital cortex; it is also directly connected to the precentral gyrus, the endpoint of the ascending nociceptive pathway. Through this CS, the IC is coupled to the DP, which is the pain‐modulating efferent limb of the network. To validate the fiber tracts as “pain‐related,” three‐dimensional masks covering the core of each tract were generated (Figure 1f). Based on these masks, the V_intra_ was extracted from each individual tract. As no headache‐ or migraine‐specific information was available for HCP subjects, DTI parameters were correlated with individual subject scores recorded by the NTPIS. In this survey, subjects were required to rate on a numerical scale of 0–10, the average level of pain they had experienced over the preceding 7 days (0 = no pain; 10 = worst pain). To assess the correlation between both parameters, a nonparametric multiple linear regression model was calculated, with the factors “age” and “gender” as covariates (see Figure 7 for summary; see Supplementary Figure S8 for exemplary data plots). After Bonferroni's correction for multiple comparisons, the left‐ and right‐sided AP showed a significant correlation with each another. Using the less conservative correction for a FDR <5%, the AP, DP, and CS tracts also significantly correlated with each other. Interestingly, there was no significant correlation between pain intensity (NTPIS) and fiber integrity (V_intra_) for the tracts in the cortical PN.

## 4 DISCUSSION

### 4.1 Three‐dimensional visualization of the migraine‐related pain signaling network

Here, we present the first synopsis of white matter tracts involved in migraine‐related pain signaling. The spatial resolution and robustness achieved by our method can be estimated from the AP: here, protopathic and epicritic fibers can be distinguished as they cross the midline at different levels of the brainstem (Figure 2, bottom). While protopathic afferents which conduct facial nociception and thermesthesia cross the midline at the level of the lower pons, epicritic fibers which conduct proprioceptive signals cross at the lemniscal decussation level of the midbrain. Furthermore, our analysis revealed that the suprasylvic part of the PCG receives particularly dense afferent input; this area contains sensory representation of head and face, and constitutes the target region of the trigeminothalamic tract (Figure 2, top). Since the fibers targeting hand and leg representation areas were also reconstructed, extratrigeminal fibers of the trigeminothalamic tract were also partially detected by the algorithm.

The structure of our matrix is conditionally defined by initial selection of the ROIs, thereby inducing a possible selection bias. However, our global tractography algorithm not only reconstructs connecting streamlines between particular ROIs, it also rebuilds the entire connectome as a whole (Reisert et al., 2011). Thus, streamline‐bundles also serve to identify regions that do belong to the network, despite not being included in the initial sample of ROIs. These regions include the auditory and visual association areas, the pre‐ and orbitofrontal cortices, and the cerebellum. The contribution of the rACC‐IC tract to the PN is characterized by an extension toward the *visual (middle occipital gyrus) and auditory (superior and medial temporal gyrus) association cortical fields* (Figure 3b). Increased activation of both regions during spontaneous human migraine attacks has previously been measured by PET imaging (Weiller et al., 1995). With respect to *visual association fields*, connections between the occipital and insular cortices were described in a previous DTI tractography study (Ghaziri et al., 2017; Menon & Uddin, 2010; Uddin et al., 2010). Approximately 90% of migraineurs report hypersensitivity to light during an attack (Wöber‐Bingöl et al., 2004) where visual cortex hyperexcitability is thought to predispose the brain to visual hypersensitivity and visual aura (Aurora, Ahmad, Welch, Bhardhwaj, & Ramadan, 1998). Furthermore, 40% of patients report that their migraines can be triggered by visual stimuli (Launer, Terwindt, & Ferrari, 1999). Thus, a structural link between the occipital cortex and the migraine network is highly plausible. Regarding the *auditory association fields*, connections between these areas and the IC have been described (Ghaziri et al., 2017), and may be involved in shifting auditory attention to focus on novel auditory stimuli (Bamiou, Musiek, & Luxon, 2003). Similar to the visual symptoms associated with migraine attacks, migraineurs frequently report sound hypersensitivity during attacks (Vingen, Pareja, Storen, White, & Stovner, 1998). As a result, noise frequently serves as a trigger of headache (Martin, Reece, & Forsyth, 2006). It is tempting to speculate that the weaker expression of the right‐sided temporal and occipital projections represent the kind of interhemispheric asymmetry known to contribute to the pathophysiology of migraine (Avnon, 2004; Crisp, Levett, Davies, Clifford Rose, & Coltheart, 1989). However, as there is no clear side‐related prevalence of migraine headache and visual symptoms (Queiroz, Friedman, Rapoport, & Purdy, 2011; Vanagaite et al., 1997), the clinical significance of this asymmetry remains obscure. Moreover, such asymmetries may also arise from artifacts in the HCP diffusion data, whose axis of susceptibility induced distortions is sagitally oriented.

The DP connects to the *pre‐ and orbitofrontal cortex in its role as* the pain modulatory efferent limb (Figure 4a,b). *Prefrontal regions* are known to be involved in emotional regulation and pain control (Brighina et al., 2011), and are targeted by noninvasive brain stimulation strategies that aim to reduce the intensity and frequency of migraine attacks (Feng, Zhang, Zhang, & Yin, 2019). Regarding the potential role of the *orbitofrontal cortex*, activation of this area is associated with habituation to painful stimuli (Weiland, Boutros, Moran, Tepley, & Bowyer, 2008). Moreover, the orbitofrontal cortex acts as a filter system for aversive inputs by actively inhibiting sensory circuits (Rule, Shimamura, & Knight, 2002). Thus, it is plausible that descending pain modulation operates under the control of frontal cortical fields.

The IC‐LC tract of the CS is wired to the ipsilateral *cerebellum* (Figure 5a). In contrast to healthy subjects, migraineurs show changes in cerebellar gray matter volume and an altered pattern of activation in response to painful trigeminal stimuli (Mehnert & May, 2019). There is also a striking comorbidity between migraine and cerebellar symptoms or diseases, and high cerebellar expression of migraine‐related genes and neuropeptides (e.g., calcitonin gene‐related peptide [CGRP]) has been reported (Kros, Angueyra Aristizábal, & Khodakhah, 2018). On a mechanistic level, the cerebellum is thought to modulate pain perception in an inhibitory manner (Ruscheweyh et al., 2014). This hypothesis is supported by our data demonstrating the spatial proximity of cerebellar connections and fibers in the DP (Figure 5c).

### 4.2 The IC: Hub of the matrix and origin of a second descending system?

By considering the entire organigram of our network (Figure 6b), the IC appears be a central interface between the ascending, processing, and descending systems. At an anatomical level, the division into a “cognitive” (i.e., anterior) insula that receives input from the anterior‐cingulate cortex and a “sensorimotor” (i.e., posterior) insula that receives input from the somatosensory cortices (Klein, Ullsperger, & Danielmeier, 2013) is corroborated by our reconstruction of the termination fields of the anterior rACC‐IC and the posterior PCG‐IC tracts (Figure 3c). Nociceptive input is initially processed in the “sensorimotor” posterior insula, where it is coded depending on pain intensity and anatomical location. The signals are then transferred to the “cognitive” anterior insula, where the emotional reaction to pain is mediated (Frot, Faillenot, & Mauguière, 2014). In line with this hypothesis, the prediction/expectation of pain occurs in the anterior insula, whereas actual pain intensity is coded within the posterior insula (Geuter, Boll, Eippert, & Büchel, 2017).

Interestingly, the insula is coupled to modulatory brainstem centers via the tracts of the CS (i.e., the IC‐LC and IC‐sTN tracts, Figure 5). Since neither of these two tracts is connected to the thalamus, it is unlikely that they are pain‐related afferents (Sherman & Guillery, 1998). Instead, they could be considered as a second descending system in addition to the “canonical” DP that is under pre‐ and orbito‐frontal control (Figure 4). This view is also in line with the hypothesis of “top‐down” (i.e., subject‐driven) modulation of nociceptive signals driven by the anterior insula under the control of the anterior cingulate cortex (for review, see (Lu et al., 2016)). This hypothesis was based both on connectivity studies in rats (Jasmin, Burkey, Granato, & Ohara, 2004) and functional imaging studies in humans (Ploner, Lee, Wiech, Bingel, & Tracey, 2011). We now provide structural evidence for fiber connections between the insula and modulatory brainstem centers in humans. In contrast to the rodent model (Jasmin et al., 2004), IC‐LC and IC‐sTN tracts originate in the posterior rather than the anterior insula. This posterior origin in humans raises the possibility that descending insula‐driven pain modulation is more related to objective pain intensity rather than the expected pain experience. However, this hypothesis requires experimental verification.

### 4.3 Linking tract anatomy to pain experience

To assess functional fiber integrity, we relied on V_intra_, which corresponds to the AWF, a dMRI parameter derived from the NODDI technique (Zhang et al., 2012). Intra‐axonal volume/water is negatively correlated to fiber damage (By et al., 2017; Margoni et al., 2019), but positively correlated to efficacious neuronal function (Genç et al., 2018). To obtain functional proof that tracked fiber bundles are indeed involved in the processing of nociceptive signals, we correlated V_intra_ with the self‐reported levels of recent pain experiences (NTPIS, (Cook et al., 2013)). The strongest correlate was found to be the ascending spino/trigeminothalamic fibers, which conduct nociceptive information to the primary somatosensory cortex. The tracts of the DP and CS showed a significant correlation with pain experience when a less conservative correction for multiple comparisons was applied. However, pain levels were not significantly correlated with the V_intra_ in the tracts of the cortical pain‐processing system. From a traditional standpoint, this cortical system (somatosensory cortices, insula, and anterior cingulum) processes different dimensions of pain experience based on a division of labor (Schnitzler & Ploner, 2000). This view was challenged by the observation that the activity of cortical pain‐processing areas is (a) not necessarily correlated with perception of pain intensity, (b) also occurs in response to nonnociceptive stimuli, and (c) is modulated by the context in which the nociceptive stimuli appear (Legrain et al., 2011). This led to an extended view of this cortical network: a system that integrates nociceptive (and other salient) stimuli in a multimodal cortical representation of the body that allows the detection and orientation toward relevant sensory events (Iannetti & Mouraux, 2010; Mouraux, Diukova, Lee, Wise, & Iannetti, 2011). The lack of a correlation between fiber integrity and individual pain experience in tracts of the cortical PN could be seen in this context. However, a pain‐specific pattern of brain activation in fMRI has been proposed based on a complex set of experimental paradigms using a machine‐learning approach (Wager et al., 2013; Zunhammer et al., 2018). Apart from the ventrolateral and medial thalamus or HT, this “neurologic pain signature” also included the anterior and dorsal insula, the secondary somatosensory cortex, and the anterior cingulate cortex, arguing for a pain‐specificity of these cortical areas. That fMRI activity of particular cortical fields may provide complementary information about network function than the integrity of tracts interconnecting these areas could explain these apparently opposing findings.

### 4.4 Migraine pathophysiology beyond white matter tracts

Migraine has been termed to be a disorder of the central nervous system (Goadsby et al., 2017) because (a) premonitory symptoms may precede headache for 2 days and are represented by changes in hypothalamic activation (Giffin et al., 2003; Schulte et al., 2020). (b) Migraine is frequently triggered by central factors such as sleep, food deprivation, and stress. (c) Migraine also comprises nonnociceptive neurological symptoms (aura, vegetative symptoms). (d) There is a cycling susceptibility of generating migraine attacks (May, 2017; Stankewitz et al., 2011) pointing to a pathophysiological process within the central nervous system. (e) Migraine is characterized by a multimodal hypersensitivity to sensory inputs that may persist in the interictal state and may be also accompanied by cortical hyperexcitability (Schwedt, 2013). Due to a dysfunction in the network of brain stem centers (e.g., PAG, RVM, and LC) and hypothalamic nuclei, a defective descending control of trigeminovascular nociceptive signaling is thought to induce a hypersensitization leading to an abnormal activation of sensory systems even under normal conditions (Goadsby et al., 2017). Our model of white matter tracts spanning through the entire brain (Figure 6) supports the idea of migraine as a central nervous system disorder.

However, our model necessarily neglects the extracerebral factors involved in migraine pathophysiology such as the processes at the neurovascular junction. Intracranial (meningeal) blood vessels are innervated by nociceptive fibers originating in the trigeminal ganglion and the C1‐3 dorsal root ganglion (Bernstein & Burstein, 2012; Moskowitz & Macfarlane, 1993). Due to release of endogenous inflammatory mediators (e.g., substance P, neurokinin A, CGRP (Holzer, 1988), a sterile “neurogenic” inflammation leads to an activation and sensitization of peripheral nociceptors and central nociceptive neurons (Bernstein & Burstein, 2012; Reuter et al., 2001). In line with this hypothesis, endogenous inflammatory mediators like substance P and the CGRP are also potent triggers of migraine (Headache Classification Committee of the International Headache Society (IHS), 2018).

Whereas our tracking approach is focused on the visualization of white matter tracts harboring the flow of pain‐related signals, various pathophysiological aspects can be only fully understood by the interplay of neurotransmitter, neuropeptides, and hormones within the system (Goadsby et al., 2017). On the level of the neurovascular junction, vasoactive neuropeptides (see above) are from particular importance as they are involved in the activation and sensitization of peripheral nociceptors and central nociceptive neurons (Bernstein & Burstein, 2012; Reuter et al., 2001). Regarding the descending modulation of trigeminovascular nociceptive transmission, serotonin, and endocannabinoids are critically involved to control the nociceptive transmission at the level of the trigeminal nucleus (Akerman et al., 2013; Humphrey & Goadsby, 1994). With respect to brainstem and hypothalamic nuclei, neuropeptides such as neuropeptide Y, leptin, or orexins have to be taken into account, that are involved in homeostatic processes (food intake and sleep regulation; (Goadsby et al., 2017)).The occurrence of these signaling systems within the core regions of migraine pathophysiology explains that sleep, food deprivation and stress are frequent trigger factors of migraine attacks.

### 4.5 Outlook and future research

Although the subjects included in this study were young and the pain levels were expectedly low (mean ± *SD*, age: 29 ± 3.7 years; NTPIS‐score: 1.63 ± 1.85), we were able to detect significant correlations between V_intra_ and pain experience for a large part of our network. Thus, application of the V_intra_ measurement within our tracts appears to be a very sensitive tool for detecting the “footprints” of recent pain experiences within the human brain. In contrast to the neurite orientation dispersion and density imaging (NODDI) approach (Zhang et al., 2012), extraction of mesoscopic diffusivity parameters with our Bayesian approach only takes about 6 min, allowing its potential application in daily clinical routine (Reisert et al., 2017). This provides the basis for investigating the power of using functional fiber integrity to monitor therapeutic effects or prognostic appraisals in migraine patients.

### 4.6 Limitations of our study

We have chosen migraine as an access path to enchase the pain‐related network as it ranges among the most well‐investigated pain disorders (Charles, 2018). Thus, we might have missed or neglected regions or connections that are involved in other pain disorders. We are aware that the migraine‐related part of the pain‐signaling network may display only a part of the entire pain‐related system of the brain. Our aim was to visualize a rough scaffold that harbors the flow of nociceptive signals, rather than carrying out an extensive structural connectivity analysis or an exhaustive review of brain regions related to pain/migraine pathophysiology. It was furthermore not the scope of this study to assess the involvement of or network in migraine patients and in particular conditions of migraine. This will be the purpose of further studies. We are furthermore aware of the limitation that the NTPIS is unspecific and does not distinguish between different modalities, qualities, and localization of pain. Thus, we cannot claim that our network data are functionally specific to any type of pain. As a consequence of this approach, further considerations should also be applied to the interpretation of our results:

1. Tracked streamlines do not denote the directionality of projections, thus the denomination of “ascending” or “descending” pathways were chosen with respect to the context.
2. It is not possible to distinguish between whether a synaptic connection occurs within a ROI, or if a streamline simply passes through it.
3. Since a threshold was applied to the probability of streamline occurrence, tracts with high individual variability in tract anatomy, or a low absolute streamline number, were omitted from our analysis. Therefore, the absence of a particular tract does not exclude the existence of functionally relevant connections between ROIs.

## CONFLICT OF INTERESTS

The authors declare no conflict of interests.

## ETHICS STATEMENT

All procedures performed in studies involving human participants were in accordance with the ethical standards of the institutional and/or national research committee and with the 1964 Helsinki declaration and its later amendments or comparable ethical standards. For more information, we refer to the HCP homepage (https://www.humanconnectome.org).

## Supporting information


**Supplementary Figure S1** **The ascending pathway (AP):** The AP main trunk in MNI space superimposed onto a T1w template (upper panel–axial; lower panel–coronal). Color‐coding in red (left) and blue (right) indicates the probability of occurrence of fiber streamlines in the entire group (in %). Yellow shading denotes the postcentral gyrus (PCG)

**Supplementary Figure S2** **The processing network (PN)—PCG‐IC tract:** Fiber connections between the PCG and insula in MNI space superimposed onto a T1w template (upper panel–axial; lower panel–coronal). Color‐coding in red (left) and blue (right) indicates the probability of occurrence of fiber streamlines in the entire group (in %). Yellow shading denotes the postcentral gyrus (PCG), green shading denotes the insular cortex

**Supplementary Figure S3** **The processing network (PN)—rACC‐IC tract:** Fiber connections between the rACC and insula in MNI space superimposed onto a T1w template (upper panel–axial; lower panel–coronal). Color‐coding in red (left) and blue (right) indicates the probability of occurrence of fiber streamlines in the entire group (in %). Magenta shading denotes the rACC, green shading denotes the insular cortex

**Supplementary Figure S4** **The descending pathway—dorsal column:** Fiber connections between all DP‐ROIs except the rostral ventromedial medulla (see Figure 1c) are indicated in MNI space superimposed onto a T1w template (upper panel–axial; lower panel–coronal). Color‐coding in red (left) and blue (right) indicates the probability of occurrence of fiber streamlines in the entire group (in %)

**Supplementary Figure S5** **The descending pathway—ventral column:** In addition to the dorsal part of the DP (red), as indicated in Supplementary Figure SS4, an unpaired ventral column (blue) can be superimposed onto MNI space that is overlaid onto a T1w template. This ventral column connects the rostral ventromedial medulla with the periaqueductal gray (PAG), without passing through the locus coeruleus. Above the level of the PAG, both columns of the DP are inseparably intermingled (magenta). Color‐coding indicates the probability of occurrence of fiber streamlines in the entire group (in %)

**Supplementary Figure S6** **The connecting system (CS)‐ IC‐LC tract:** Fiber connections between the IC and LC in MNI space superimposed onto a T1w template (upper panel–axial; lower panel–coronal). Color‐coding in red (left) and blue (right) indicates the probability of occurrence of fiber streamlines in the entire group (in %). Green shading denotes the insular cortex

**Supplementary Figure S7** **The connecting system (CS)‐ IC‐sTN tract:** Fiber connections between the IC and sTN in MNI space superimposed onto a T1w template (upper panel–axial; lower panel–coronal). Color‐coding in red (left) and blue (right) indicates the probability of occurrence of fiber streamlines in the entire group (in %). Green shading denotes the insular cortex

**Supplementary Figure S8** **Correlation of functional fiber integrity with self‐reported pain levels:** Self‐reported pain levels assessed by the NIH Toolbox Pain Intensity Survey (NTPIS) were correlated with V_intra_ using a non‐parametric multiple linear regression model. Raw data are plotted for the four tracts, showing the highest correlations. Ascending pathway (AP), descending pathway (DP), connecting system (CS)

**Supplementary Table S1** **Results of the literature search carried out to identify migraine‐associated ROIs. ACC:** anterior cingulate cortex; **DLP:** dorsolateral pons; **dlPFC:** dorsolateral prefrontal cortex; **FG:** fusiform gyrus; **fMRI:** functional magnetic resonance imaging; **Hippo:** hippocampus; **HT:** hypothalamus; **IC:** insular cortex; **LC**: locus coeruleus; **LN:** lentiform nucleus; **M1:** precentral gyrus; **MCC:** middle cingulate cortex; **MDH:** medullary dorsal horn; **mPFC:** medial prefrontal cortex; **OFC:** orbitofrontal cortex; **OR:** original research; **PAG:** periaqueductal gray; **PET:** positron emission tomography; **PB:** parabrachial nucleus; **PFC:** prefrontal cortex; **RetN:** reticular nucleus; **Rev:** review article; **RVM:** rostral ventromedial medulla; **S1:** primary somatosensory cortex; **S2:** secondary somatosensory cortex; **sTN:** spinal trigeminal nucleus; **TH:** thalamus; **TN:** trigeminal nucleus; **V1:** primary visual cortex; **V2:** secondary visual cortex.
**Supplementary Table S2: Results of the literature search carried out to identify general pain‐associated ROIs. ACC:** anterior cingulate cortex; **Amyg:** amygdala; **CfN:** cuneiform nucleus; **dlPFC:** dorsolateral prefrontal cortex; **fMRI:** functional magnetic resonance imaging; **HT:** hypothalamus; **IC:** insular cortex; **LC**: locus coeruleus; **mPFC:** medial prefrontal cortex; **OR:** original research; **PAG:** periaqueductal gray; **PET:** positron emission tomography; **PB:** parabrachial nucleus; **RetN:** reticular nucleus; **Rev:** review article; **RVM:** rostral ventromedial medulla; **S1:** primary somatosensory cortex; **S2:** secondary somatosensory cortex; **sTN:** spinal trigeminal nucleus; **TH:** thalamus
**Supplementary Table S3: Threshold values used for the creation of tract masks.** Threshold values were defined based on probability maps. Only voxels with a certain probability of streamline occurrence (i.e., “lower limit”) were taken into account. The “upper limit” was defined by the voxel showing the maximal probability of streamline occurrence

## Data Availability

Data and code are available from the authors upon reasonable request.
