# Supplementary figures and images for: Approximation to pain‐signaling network in humans by means of migraine
Source: Hum Brain Mapp. 2020 Oct 28;42(3):766–79. doi: 10.1002/hbm.25261 (PMC7814755; doi:10.1002/hbm.25261)

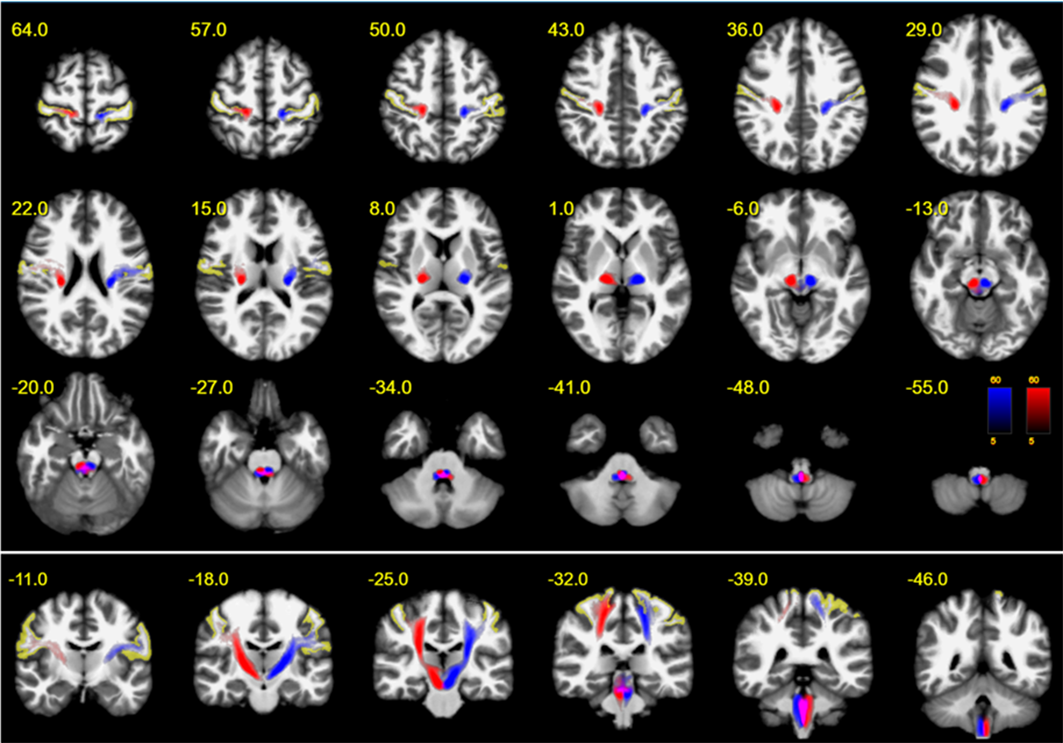

Supplement: Supplementary file 1 — Supplementary Figure S1 The ascending pathway (AP): The AP main trunk in MNI space superimposed onto a T1w template (upper panel–axial; lower panel–coronal). Color‐coding in red (left) and blue (right) indicates the probability of occurrence of fiber streamlines in the entire group (in %). Yellow shading denotes the postcentral gyrus (PCG) [file HBM-42-766-s001.tif]

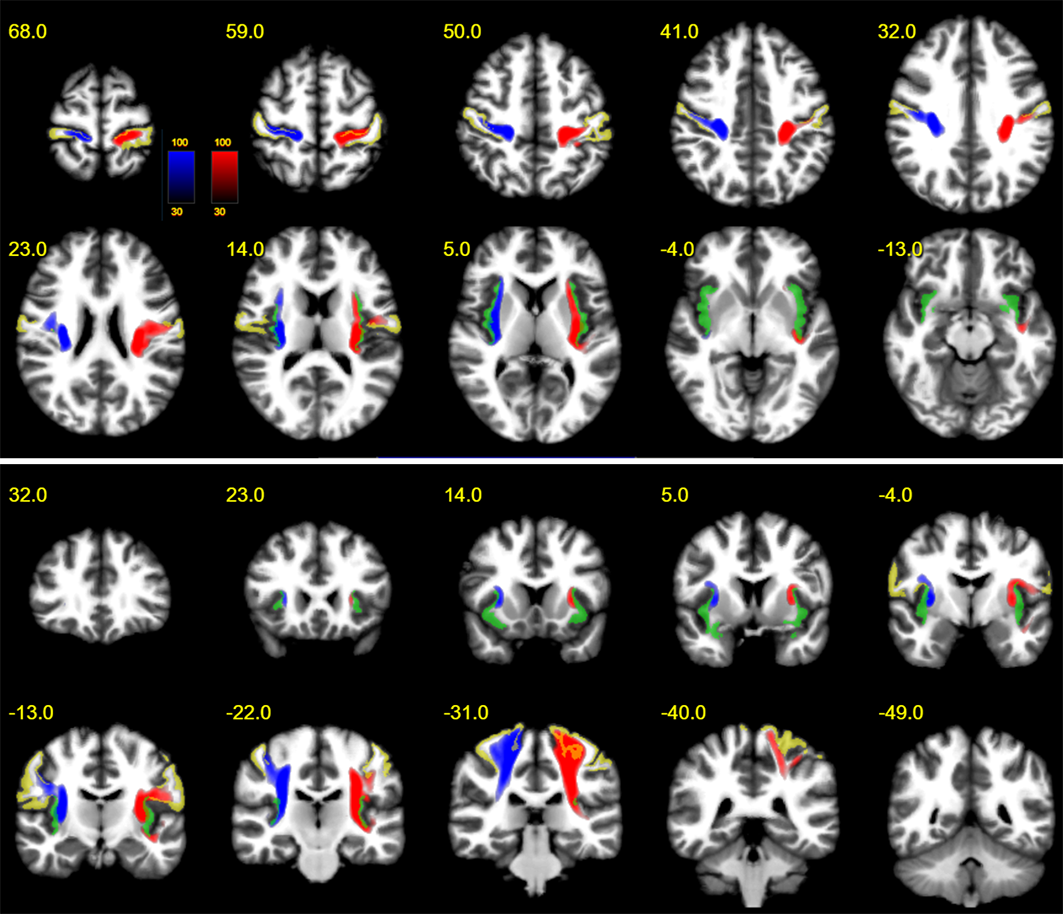

Supplement: Supplementary file 2 — Supplementary Figure S2 The processing network (PN)—PCG‐IC tract: Fiber connections between the PCG and insula in MNI space superimposed onto a T1w template (upper panel–axial; lower panel–coronal). Color‐coding in red (left) and blue (right) indicates the probability of occurrence of fiber streamlines in the entire group (in %). Yellow shading denotes the postcentral gyrus (PCG), green shading denotes the insular cortex [file HBM-42-766-s002.tif]

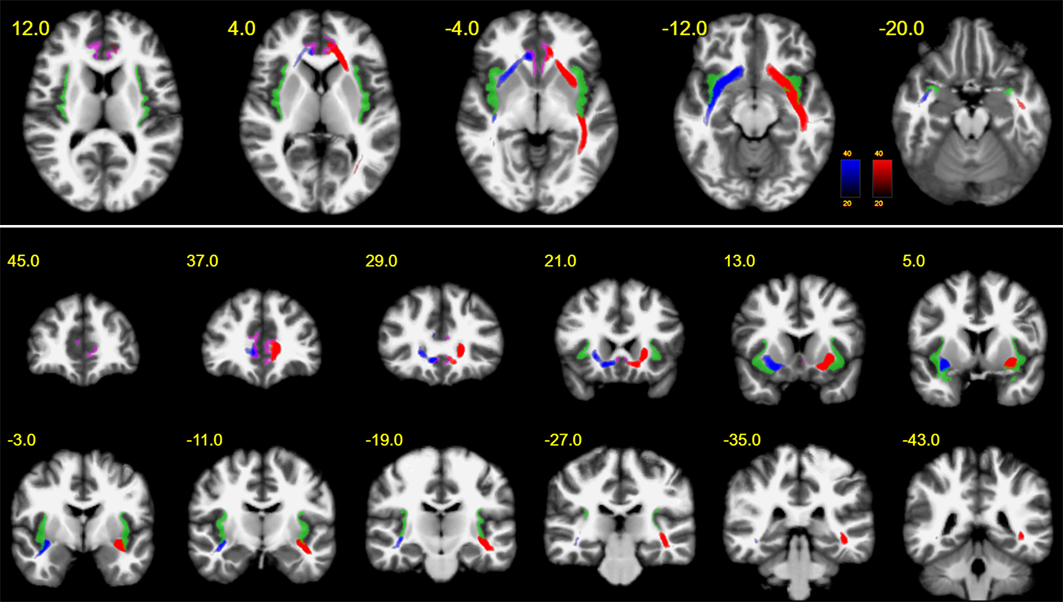

Supplement: Supplementary file 3 — Supplementary Figure S3 The processing network (PN)—rACC‐IC tract: Fiber connections between the rACC and insula in MNI space superimposed onto a T1w template (upper panel–axial; lower panel–coronal). Color‐coding in red (left) and blue (right) indicates the probability of occurrence of fiber streamlines in the entire group (in %). Magenta shading denotes the rACC, green shading denotes the insular cortex [file HBM-42-766-s003.tif]

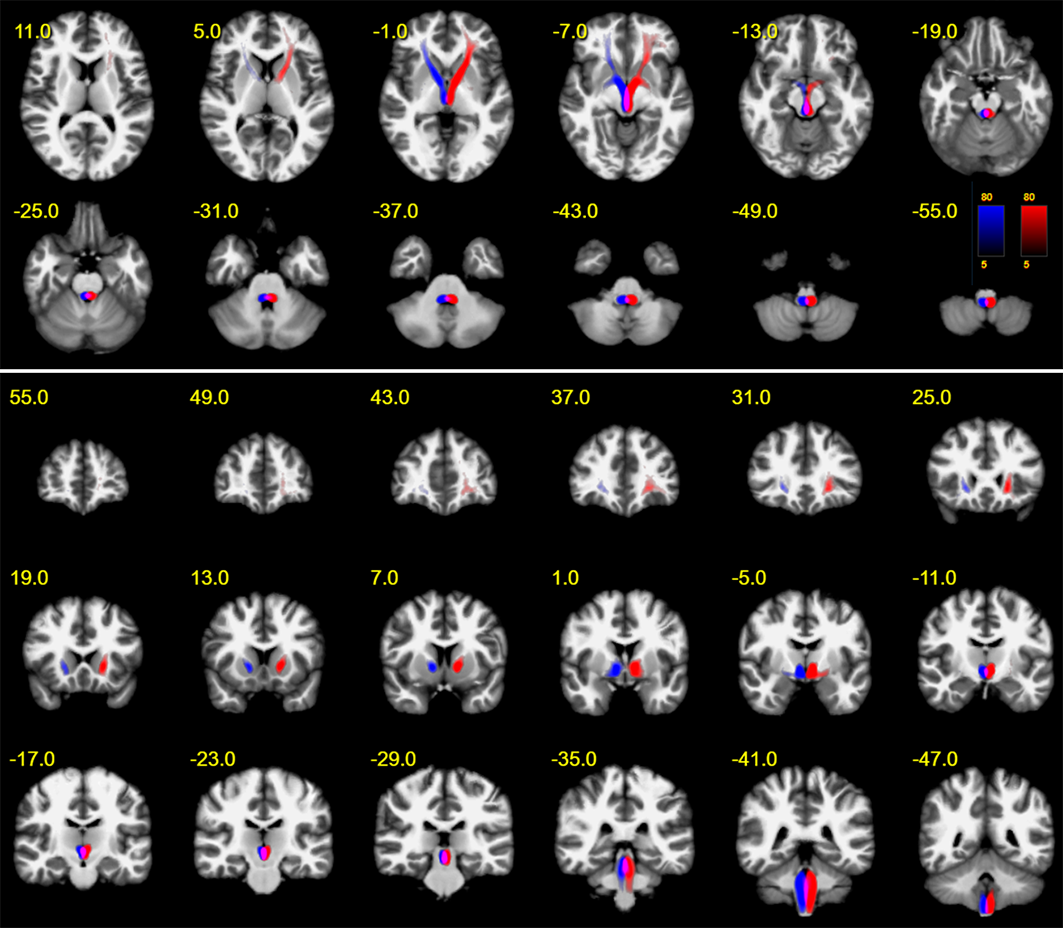

Supplement: Supplementary file 4 — Supplementary Figure S4 The descending pathway—dorsal column: Fiber connections between all DP‐ROIs except the rostral ventromedial medulla (see Figure 1c) are indicated in MNI space superimposed onto a T1w template (upper panel–axial; lower panel–coronal). Color‐coding in red (left) and blue (right) indicates the probability of occurrence of fiber streamlines in the entire group (in %) [file HBM-42-766-s004.tif]

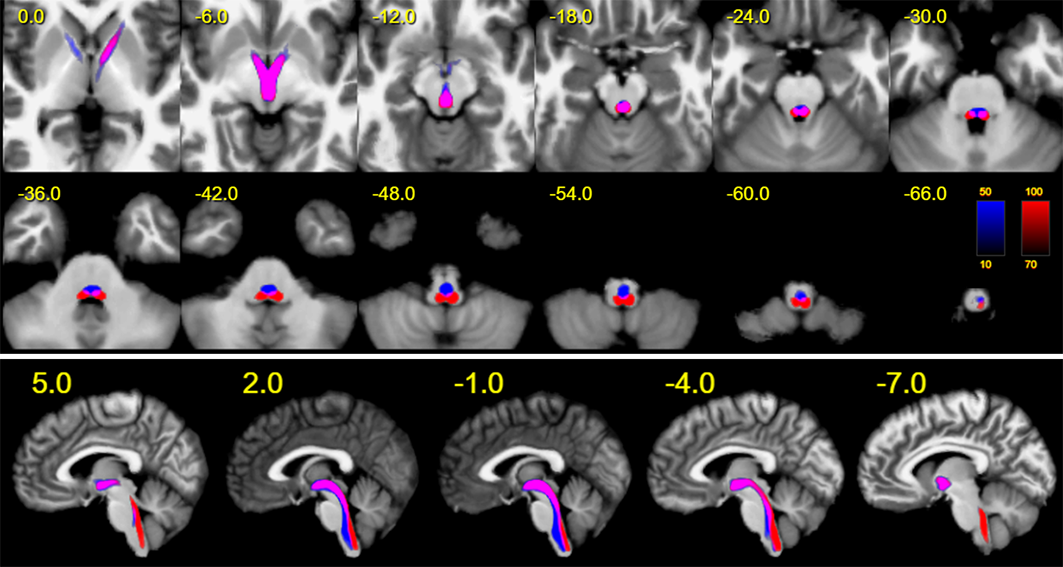

Supplement: Supplementary file 5 — Supplementary Figure S5 The descending pathway—ventral column: In addition to the dorsal part of the DP (red), as indicated in Supplementary Figure SS4, an unpaired ventral column (blue) can be superimposed onto MNI space that is overlaid onto a T1w template. This ventral column connects the rostral ventromedial medulla with the periaqueductal gray (PAG), without passing through the locus coeruleus. Above the level of the PAG, both columns of the DP are inseparably intermingled (magenta). Color‐coding indicates the probability of occurrence of fiber streamlines in the entire group (in %) [file HBM-42-766-s005.tif]

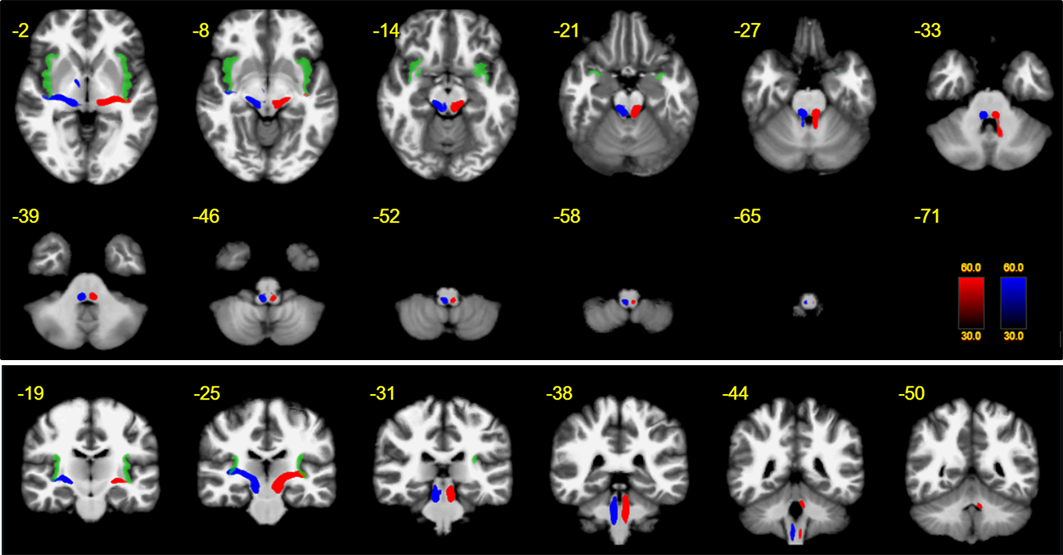

Supplement: Supplementary file 6 — Supplementary Figure S6 The connecting system (CS)‐ IC‐LC tract: Fiber connections between the IC and LC in MNI space superimposed onto a T1w template (upper panel–axial; lower panel–coronal). Color‐coding in red (left) and blue (right) indicates the probability of occurrence of fiber streamlines in the entire group (in %). Green shading denotes the insular cortex [file HBM-42-766-s006.tif]

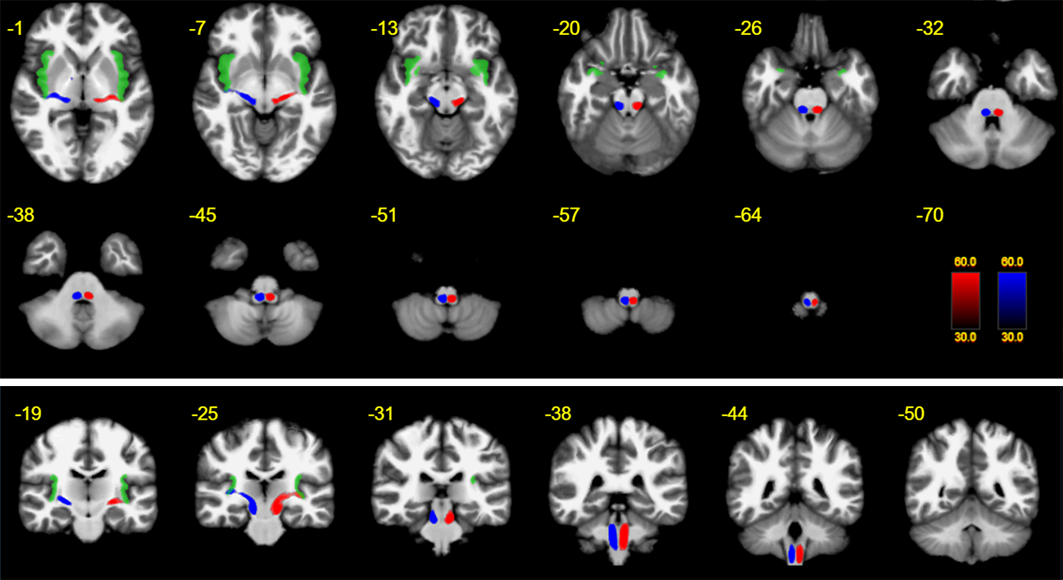

Supplement: Supplementary file 7 — Supplementary Figure S7 The connecting system (CS)‐ IC‐sTN tract: Fiber connections between the IC and sTN in MNI space superimposed onto a T1w template (upper panel–axial; lower panel–coronal). Color‐coding in red (left) and blue (right) indicates the probability of occurrence of fiber streamlines in the entire group (in %). Green shading denotes the insular cortex [file HBM-42-766-s007.tif]

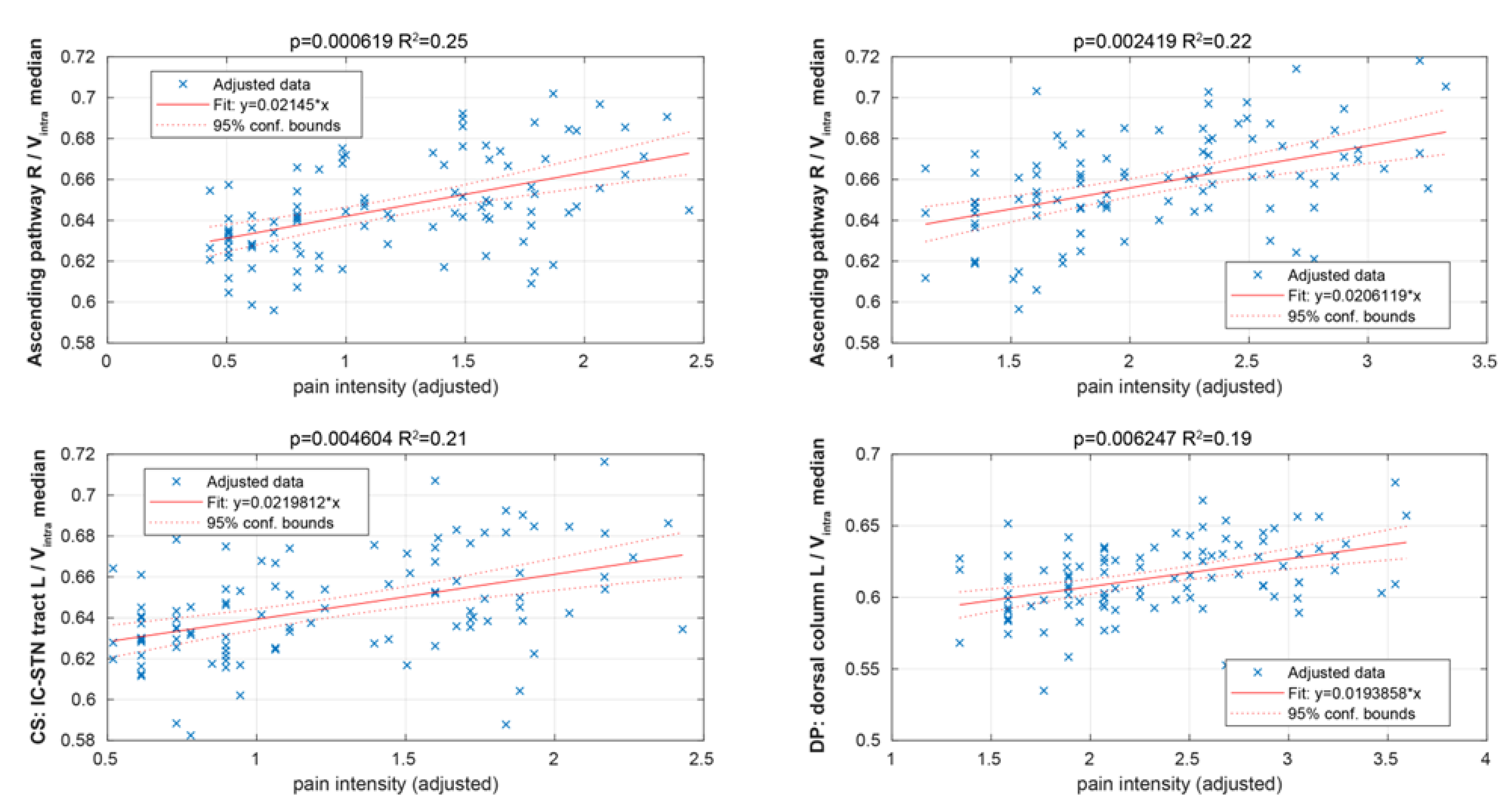

Supplement: Supplementary file 8 — Supplementary Figure S8 Correlation of functional fiber integrity with self‐reported pain levels: Self‐reported pain levels assessed by the NIH Toolbox Pain Intensity Survey (NTPIS) were correlated with Vintra using a non‐parametric multiple linear regression model. Raw data are plotted for the four tracts, showing the highest correlations. Ascending pathway (AP), descending pathway (DP), connecting system (CS) [file HBM-42-766-s008.tif]
