# Supplementary material for: Approximation to pain‐signaling network in humans by means of migraine
Source: Hum Brain Mapp. 2020 Oct 28;42(3):766–79. doi: 10.1002/hbm.25261 (PMC7814755; doi:10.1002/hbm.25261)
Supplement: Supplementary file 9 — Supplementary Table S1 Results of the literature search carried out to identify migraine‐associated ROIs. ACC: anterior cingulate cortex; DLP: dorsolateral pons; dlPFC: dorsolateral prefrontal cortex; FG: fusiform gyrus; fMRI: functional magnetic resonance imaging; Hippo: hippocampus; HT: hypothalamus; IC: insular cortex; LC: locus coeruleus; LN: lentiform nucleus; M1: precentral gyrus; MCC: middle cingulate cortex; MDH: medullary dorsal horn; mPFC: medial prefrontal cortex; OFC: orbitofrontal cortex; OR: original research; PAG: periaqueductal gray; PET: positron emission tomography; PB: parabrachial nucleus; PFC: prefrontal cortex; RetN: reticular nucleus; Rev: review article; RVM: rostral ventromedial medulla; S1: primary somatosensory cortex; S2: secondary somatosensory cortex; sTN: spinal trigeminal nucleus; TH: thalamus; TN: trigeminal nucleus; V1: primary visual cortex; V2: secondary visual cortex. Supplementary Table S2: Results of the literature search carried out to identify general pain‐associated ROIs. ACC: anterior cingulate cortex; Amyg: amygdala; CfN: cuneiform nucleus; dlPFC: dorsolateral prefrontal cortex; fMRI: functional magnetic resonance imaging; HT: hypothalamus; IC: insular cortex; LC: locus coeruleus; mPFC: medial prefrontal cortex; OR: original research; PAG: periaqueductal gray; PET: positron emission tomography; PB: parabrachial nucleus; RetN: reticular nucleus; Rev: review article; RVM: rostral ventromedial medulla; S1: primary somatosensory cortex; S2: secondary somatosensory cortex; sTN: spinal trigeminal nucleus; TH: thalamus Supplementary Table S3: Threshold values used for the creation of tract masks. Threshold values were defined based on probability maps. Only voxels with a certain probability of streamline occurrence (i.e., “lower limit”) were taken into account. The “upper limit” was defined by the voxel showing the maximal probability of streamline occurrence [file HBM-42-766-s009.docx]

**Supplementary Tables**

| **Species** | **Ref.** | **Method** | **Regions involved** | **Type** | **Model** | **Comment** |
| --- | --- | --- | --- | --- | --- | --- |
| Humans | ^1^ | fMRI | sTN, HT | OR | Migraine without aura, ictal, peri-ictal ad inter-ictal | Increased activation of HT preceding migraine attack; altered functional coupling between HT and sTN |
| Humans | ^2^ | PET | brainstem, cingulate, auditory and visual cortices | OR | Migraine without aura, 6h after attack | Brainstem is involved in triggering spontaneous migraine attacks |
| Humans, Animals | ^3^ | Mult. methods covered | TN, RVM, LC, S1, S2  HT, TH, IC, PAG, V1, V2 | Rev | All aspects of migraine covered | Exhaustive review covering all aspects of migraine pathophysiology |
| Humans | ^4^ | resting-state fMRI | PAG, ACC, PFC, S1 | OR | Migraine with and without aura, during a pain‐free state | Impairment of the descending pain modulatory circuits in migraine |
| Rodents | ^5^ | Pharmacology, Electrophysiology | RVM | OR | Male healthy Sprague-Dawley rats, application of inflammatory mediators into RVM (migraine model) | Pain-facilitating neurons in the RVM mediate headache-related pain |
| Rodents | ^6^ | fMRI,  Electrophysiology | rACC | OR | Male healthy Sprague-Dawley rats, inflammatory soup infusion (migraine model) | Altered connectivity between rACC to other brain regions in a rat model of migraine |
| Humans | ^7^ | fMRI | DLP, RVM, LN, S1, ACC, MCC, dlPFC, Hippo, FG, M1 | Rev | Migraine in various conditions, ictal, peri-ictal and inter-ictal | Summary of activation patterns revealed by fMRI in different migraine conditions regarding the phenomenon of sensory hypersensitivity |
| Humans | ^8^ | Mult. methods covered | sTN, HT, PAG, RVM, TH | Rev | Migraine, not specified | Summary of the anatomy and sensory physiology of migraine headache, focus on neurovascular/periphery physiology |
| Humans | ^9^ | fMRI, PET | IC | Rev | Migraine in various conditions | Role of IC in the physiology of migraine |
| Humans | ^10^ | fMRI, PET | TN, PAG, DLP (LC, RetN, PbN), TH (post. nuclei), S1 | Rev | Migraine in various conditions | Summary of the anatomy and sensory physiology of migraine headache |
| Humans, Animals | ^11^ | Mult. methods covered | sTN, PAG, RVM, HT, TH, S1, IC | Rev | Migraine in various conditions | Summary of the anatomy and sensory physiology of migraine headache |
| Humans, Animals | ^12^ | Mult. methods covered | sTN, TH, MDH, PAG, RVM | Rev | Migraine, not specified | Summary of the anatomy and sensory physiology of migraine headache, focus on neurovascular/periphery physiology |
| Humans, Animals | ^13^ | Mult. methods covered | sTN, PAG. RVM, HT, TH (post. nuclei), S1, S2, IC, M1, M2 | Rev | Migraine in various conditions | Summary of the anatomy and physiology of migraine |

**Supplementary Table 1: Results of the literature search carried out to identify migraine-associated ROIs. ACC:** anterior cingulate cortex; **DLP:** dorsolateral pons; **dlPFC:** dorsolateral prefrontal cortex; **FG:** fusiform gyrus; **fMRI:** functional magnetic resonance imaging; **Hippo:** hippocampus; **HT:** hypothalamus; **IC:** insular cortex; **LC**: locus coeruleus; **LN:** lentiform nucleus; **M1:** precentral gyrus; **MCC:** middle cingulate cortex; **MDH:** medullary dorsal horn; **mPFC:** medial prefrontal cortex; **OFC:** orbitofrontal cortex; **OR:** original research; **PAG:** periaqueductal grey; **PET:** positron emission tomography; **PB:** parabrachial nucleus; **PFC:** prefrontal cortex; **RetN:** reticular nucleus; **Rev:** review article; **RVM:** rostral ventromedial medulla; **S1:** primary somatosensory cortex; **S2:** secondary somatosensory cortex; **sTN:** spinal trigeminal nucleus; **TH:** thalamus; **TN:** trigeminal nucleus; **V1:** primary visual cortex; **V2:** secondary visual cortex.

| **Species** | **Ref** | **Method** | **Region involved** | **Type** | **Model** | **Comment** |
| --- | --- | --- | --- | --- | --- | --- |
| Humans | ^14^ | fMRI | sTN, LC, CfN, RVM, PAG, HT, TH, S2, IC | OR | Trigeminal noxious stimulus, healthy subjects | Activation pattern after application of a trigeminal noxious stimulus |
| Humans | ^15^ | fMRI | IC | OR | Cutaneous heat stimuli, healthy subjects | Insula as central pain-processing region |
| Humans | ^16^ | perfusion MRI | dorsal IC | OR | Topical capsaicin cream, healthy subjects | CBF in the dorsal IC correlates to pain intensity |
| Humans | ^17^ | fMRI | ACC, S1, S2, IC | Rev | Multimodal stimuli, healthy subjects | Cortical pain-processing areas act as a system detecting salient sensory events. |
| Humans, Animals | ^18^ | Mult. methods covered | PAG, PB, RVM, LC, Amyg, TH, S1, mPFC, ACC, IC | Rev | General pain | Summary of the anatomy and physiology of pain processing |
| Humans, Animals | ^19^ | Mult. methods covered | ACC, S1, S2, IC | Rev | General pain | Summary of the anatomy and physiology of pain processing |
| Rodents | ^20^ | Mult. methods covered | RVM, RetN, PAG, HT, ACC, mPFC, Amyg | Rev | General pain | Summary of pain modulation by the descending system |

**Supplementary Table 2: Results of the literature search carried out to identify general pain-associated ROIs. ACC:** anterior cingulate cortex; **Amyg:** amygdala; **CfN:** cuneiform nucleus; **dlPFC:** dorsolateral prefrontal cortex; **fMRI:** functional magnetic resonance imaging; **HT:** hypothalamus; **IC:** insular cortex; **LC**: locus coeruleus; **mPFC:** medial prefrontal cortex; **OR:** original research; **PAG:** periaqueductal grey; **PET:** positron emission tomography; **PB:** parabrachial nucleus; **RetN:** reticular nucleus; **Rev:** review article; **RVM:** rostral ventromedial medulla; **S1:** primary somatosensory cortex; **S2:** secondary somatosensory cortex; **sTN:** spinal trigeminal nucleus; **TH:** thalamus

| **Tract** | **Lower limit (% of streamline occurrence)** | **Upper limit (% of streamline occurrence)** |
| --- | --- | --- |
| Ascending pathway (AP) | 50 | 60 |
| PG-IC tract | 80 | 100 |
| rACC-IC tract | 30 | 40 |
| Descending pathway (DP) dorsal column | 80 | 100 |
| Descending pathway (DP) ventral column | 40 | 60 |
| IC-LC tract | 30 | 60 |
| IC-sTN tract | 30 | 60 |

**Supplementary Table 3: Threshold values used for the creation of tract masks.** Threshold values were defined based on probability maps. Only voxels with a certain probability of streamline occurrence (i.e. “lower limit”) were taken into account. The “upper limit” was defined by the voxel showing the maximal probability of streamline occurrence.

**References:**

1. Schulte, L. H. & May, A. The migraine generator revisited: continuous scanning of the migraine cycle over 30 days and three spontaneous attacks. *Brain* **139**, 1987–1993 (2016).

2. Weiller, C. *et al.* Brain stem activation in spontaneous human migraine attacks. *Nat. Med.* **1**, 658–660 (1995).

3. Goadsby, P. J. *et al.* Pathophysiology of Migraine: A Disorder of Sensory Processing. *Physiol. Rev.* **97**, 553–622 (2017).

4. Mainero, C., Boshyan, J. & Hadjikhani, N. Altered functional magnetic resonance imaging resting-state connectivity in periaqueductal gray networks in migraine. *Ann Neurol.* **70**, 838–845 (2011).

5. Edelmayer, R. M. *et al.* Medullary pain facilitating neurons mediate allodynia in headache-related pain. *Ann. Neurol.* **65**, 184–193 (2009).

6. Jia, Z., Chen, X., Tang, W., Zhao, D. & Yu, S. Atypical functional connectivity between the anterior cingulate cortex and other brain regions in a rat model of recurrent headache. *Mol Pain* **15**, 174480691984248 (2019).

7. Schwedt, T. J., Chiang, C.-C., Chong, C. D. & Dodick, D. W. Functional MRI of migraine. *The Lancet Neurology* **14**, 81–91 (2015).

8. Olesen, J., Burstein, R., Ashina, M. & Tfelt-Hansen, P. Origin of pain in migraine: evidence for peripheral sensitisation. *The Lancet Neurology* **8**, 679–690 (2009).

9. Borsook, D. *et al.* The Insula: A “Hub of Activity” in Migraine. *Neuroscientist* **22**, 632–652 (2016).

10. Borsook, D. & Burstein, R. The enigma of the dorsolateral pons as a migraine generator. *Cephalalgia* **32**, 803–812 (2012).

11. Noseda, R. & Burstein, R. Migraine pathophysiology: Anatomy of the trigeminovascular pathway and associated neurological symptoms, cortical spreading depression, sensitization, and modulation of pain: *Pain* **154**, S44–S53 (2013).

12. Bernstein, C. & Burstein, R. Sensitization of the trigeminovascular pathway: perspective and implications to migraine pathophysiology. *J Clin Neurol* **8**, 89–99 (2012).

13. Pietrobon, D. & Moskowitz, M. A. Pathophysiology of Migraine. *Annu. Rev. Physiol.* **75**, 365–391 (2013).

14. Schulte, L. H., Sprenger, C. & May, A. Physiological brainstem mechanisms of trigeminal nociception: An fMRI study at 3T. *NeuroImage* **124**, 518–525 (2016).

15. Geuter, S., Boll, S., Eippert, F. & Büchel, C. Functional dissociation of stimulus intensity encoding and predictive coding of pain in the insula. *eLife* **6**, e24770 (2017).

16. Segerdahl, A. R., Mezue, M., Okell, T. W., Farrar, J. T. & Tracey, I. The dorsal posterior insula subserves a fundamental role in human pain. *Nat Neurosci* **18**, 499–500 (2015).

17. Legrain, V., Iannetti, G. D., Plaghki, L. & Mouraux, A. The pain matrix reloaded. *Progress in Neurobiology* **93**, 111–124 (2011).

18. Lu, C. *et al.* Insular Cortex is Critical for the Perception, Modulation, and Chronification of Pain. *Neurosci Bull* **32**, 191–201 (2016).

19. Schnitzler, A. & Ploner, M. Neurophysiology and functional neuroanatomy of pain perception. *J Clin Neurophysiol* **17**, 592–603 (2000).

20. Heinricher, M. M., Tavares, I., Leith, J. L. & Lumb, B. M. Descending control of nociception: Specificity, recruitment and plasticity. *Brain Research Reviews* **60**, 214–225 (2009).
